# Crosstalk between FTH1 and PYCR1 dysregulates proline metabolism and mediates cell growth in *KRAS*-mutant pancreatic cancer cells

**DOI:** 10.1038/s12276-024-01300-4

**Published:** 2024-09-18

**Authors:** Ji Min Park, Yen-Hao Su, Chi-Shuan Fan, Hsin-Hua Chen, Yuan-Kai Qiu, Li-Li Chen, Hsin-An Chen, Thamil Selvee Ramasamy, Jung-Su Chang, Shih-Yi Huang, Wun-Shaing Wayne Chang, Alan Yueh-Luen Lee, Tze-Sing Huang, Cheng-Chin Kuo, Ching-Feng Chiu

**Affiliations:** 1 Graduate Institute of Metabolism and Obesity Sciences, Taipei Medical University, Taipei, Taiwan; 2 School of Nutrition and Health Sciences, Taipei Medical University, Taipei, Taiwan; 3 Institute of Cellular and System Medicine, National Health Research Institutes, Zhunan, Taiwan; 4 Division of General Surgery, Department of Surgery, Shuang Ho Hospital, Taipei Medical University, New Taipei City, Taiwan; 5 Department of Surgery, School of Medicine, College of Medicine, Taipei Medical University, Taipei, Taiwan; 6 TMU Research Center of Cancer Translational Medicine, Taipei Medical University, Taipei, Taiwan; 7 National Institute of Cancer Research, National Health Research Institutes, Zhunan, Taiwan; 8 Stem Cell Biology Laboratory, Department of Molecular Medicine, Faculty of Medicine, Universiti Malaya, Kuala Lumpur, 50603 Malaysia; 9 Nutrition Research Center, Taipei Medical University Hospital, Taipei, Taiwan; 10 Department of Bioscience Technology, Chung Yuan Christian University, Taoyuan, Taiwan; 11 Taipei Medical University and Affiliated Hospitals Pancreatic Cancer Groups, Taipei Cancer Center, Taipei Medical University, Taipei, Taiwan

**Keywords:** Pancreatic cancer, Drug development, Diagnostic markers

## Abstract

Ferritin, comprising heavy (FTH1) and light (FTL) chains, is the main iron storage protein, and pancreatic cancer patients exhibit elevated serum ferritin levels. Specifically, higher ferritin levels are correlated with poorer pancreatic ductal adenocarcinoma (PDAC) prognosis; however, the underlying mechanism and metabolic programming of ferritin involved in *KRAS*-mutant PDAC progression remain unclear. Here, we observed a direct correlation between FTH1 expression and cell viability and clonogenicity in *KRAS*-mutant PDAC cell lines as well as with in vivo tumor growth through the control of proline metabolism. Our investigation highlights the intricate relationship between FTH1 and pyrroline-5-carboxylate reductase 1 (PYCR1), a crucial mitochondrial enzyme facilitating the glutamate-to-proline conversion, underscoring its impact on proline metabolic imbalance in *KRAS*-mutant PDAC. This regulation is further reversed by miR-5000-3p, whose dysregulation results in the disruption of proline metabolism, thereby accentuating the progression of *KRAS*-mutant PDAC. Additionally, our study demonstrated that deferasirox, an oral iron chelator, significantly diminishes cell viability and tumor growth in *KRAS*-mutant PDAC by targeting FTH1-mediated pathways and altering the PYCR1/PRODH expression ratio. These findings underscore the novel role of FTH1 in proline metabolism and its potential as a target for PDAC therapy development.

## Elevated ferritin levels drive poor prognosis in pancreatic cancer

Iron storage in our body is mainly controlled by a protein named ferritin, which reflects the amount of stored iron through its blood levels. Low ferritin levels usually suggest iron-deficiency anemia, while high levels can indicate inflammation and hinting at ferritin’s potential as a cancer indicator. In this study, scientists focused on pancreatic cancer, notorious for its low survival rates and limited treatment options. They examined the expression of different ferritin components and their link with the KRAS mutation, a common characteristic in pancreatic cancer that promotes tumor growth. The main discovery is that high FTH1 expression is associated with worse survival in pancreatic cancer patients, suggesting that targeting FTH1 could be a promising treatment for this aggressive cancer. This study enhances our knowledge of the molecular processes driving pancreatic cancer and opens new paths for targeted treatments. This summary was initially drafted using artificial intelligence, then revised and fact-checked by the author.

## Introduction

Ferritin is a protein primarily known for its central role in iron storage; the serum ferritin (SF) level is positively correlated with the amount of iron stored within the body systemically. The normal SF range is 30–300 ng/mL in men and 10–200 ng/mL in women; however, individuals with iron-deficiency anemia tend to demonstrate relatively low SF levels, whereas those with chronic and acute inflammation exhibit relatively high SF levels^1,2^. Epidemiological studies have demonstrated that SF levels may be used as a predictive biomarker for various cancers, including hepatocellular, lung, and breast cancers. In these cancers, the greater the severity is, the higher the SF levels are; as such, increasing SF levels are correlated with worsening cancer survival^3–5^. This correlation cannot be explained only by patient inflammatory status: even after adjustments for inflammatory markers in a multivariable model, a negative association between SF and cancer survival was still noted^6^. The prognostic value of SF in various cancers has been examined to some extent; however, the function and regulation of ferritin in tumor progression and its therapeutic potential remain to be further investigated.

Although the source of SF is unclear, several studies have suggested that secreted ferritin contains both heavy (FTH1) and light (FTL) ferritin chain subunits, and its subunit composition in tumor ferritins may vary among different cancers^1,7^. In patients with breast cancer, for instance, increases in SF are strongly correlated with FTL rather than with FTH1, whereas these increases may be due to FTH1 overexpression in patients with acute myeloid leukemia (AML). In other words, the expression patterns and functional roles of FTH1 and FTL are distinguishable based on the cancer type^8–10^. Moreover, the different *FTH1*/*FTL* ratios within a single ferritin complex may contribute to tumorigenesis in many malignancies^1^. Thus, investigating ferritin subunit expression and distribution within specific tumor cells may provide further insight into the roles of ferritin in tumor development and progression.

Pancreatic cancer has the highest mortality rate of all major cancers. It is the third leading cause of cancer death in the United States with the lowest 5-year survival rate among all cancers (~11%) in 2022^11^. Currently, pancreatic cancer incidence and mortality are nearly equivalent because of the lack of reliable pancreatic cancer biomarkers and treatment options available. Pancreatic ductal adenocarcinoma (PDAC) is the most common type of pancreatic cancer, and nearly 95% of patients with PDAC harbor a *KRAS* mutation. *KRAS* is widely known as the critical driver that enables unlimited proliferation, apoptosis resistance, and metastasis in pancreatic cancer cells and promotes metabolic alterations for the sustenance of biosynthetic pathways. Although *KRAS* is one of the most well-known protooncogenes, reliable *KRAS*-targeted anticancer strategies have not been reported thus far; moreover, *KRAS* mutations are considered undruggable targets^12,13^.

We previously reported a case–control Taiwanese cohort study that investigated the association between high SF and pancreatic cancer risk, along with a relevant meta-analysis, emphasizing the need to explore the involvement of ferritin in pancreatic cancer progression^14^. The present study was designed to explore the expression of ferritin subunits and *KRAS* mutation status in pancreatic cancer. We specifically investigated whether FTH1 or FTL is involved in the regulation of *KRAS*-mutant PDAC cell growth and metabolic reprogramming. Notably, we found that FTH1 is strongly expressed in PDAC harboring a *KRAS* mutation and contributes to proline metabolism reprogramming through crosstalk with pyrroline-5-carboxylate reductase 1 (PYCR1). Deferasirox (DFX), an iron chelator, was also found to have an antiproliferative effect on pancreatic cancer cells via the suppression of FTH1 expression, suggesting that FTH1 expression or activity may be exploited as an effective therapeutic tool to target *KRAS*-mutant PDAC.

## Materials and methods

### Cell culture

Human nonmalignant pancreatic epithelial (hTERT-HPNE) and human PDAC [BxPC-3, AsPC-1, Mia PaCa-2, SUIT-2, PANC-1, and PANC-1/gemcitabine resistance (GR)] cell lines were kindly provided by Drs. Wun-Shaing Wayne Chang and Li-Tzong Chen from the National Institute of Cancer Research (NHRI, Taiwan) and the human embryonic kidney cell line HEK293T was purchased from the Bioresource Collection and Research Center (Hsinchu, Taiwan). The hTERT-HPNE cells were cultured in Dulbecco’s modified Eagle’s medium (DMEM) low glucose containing 5% fetal bovine serum (FBS), 1% penicillin–streptomycin (PS), and 10 ng/mL epidermal growth factor (EGF). BxPC-3, PANC-1, PANC-1/GR, SUIT-2, and AsPC-1 cells were maintained in Roswell Park Memorial Institute (RPMI) 1640 medium supplemented with 10% FBS and 1% PS. Finally, the Mia PaCa-2 cells were maintained in high-glucose DMEM supplemented with 10% FBS, 1% PS, and 2.5% horse serum. DesPanc03, a primary mouse pancreatic cancer cell line, was established from a 12-month-old *LSL-Kras*^*G12D*^*/Pdx1*^*cre*^ (KC) mouse exhibiting a highly fibrotic form of pancreatic ductal adenocarcinoma (PDAC). To ensure optimal growth and viability, the cells were cultured in high-glucose DMEM supplemented with 10% FBS and 1% PSG (#10378016, Thermo). This medium composition provides the necessary nutrients and growth factors, along with protection against bacterial contamination, facilitating the maintenance of the cell line under standard cell culture conditions. The cells were cultured at 37 °C and 5% CO_2_ in a humidified incubator. The strains were verified to be mycoplasma free, and their identities were authenticated through short tandem repeat (STR) profiling conducted by both the Bioresource Collection and Research Center (Hsinchu, Taiwan) and the Center for Genomic Medicine at the National Center for Neoliberal Medicine (NCKU) (Tainan, Taiwan). The culture media were routinely refreshed every three days to maintain optimal growth conditions, and the cultures were allowed to reach 80–90% confluency before proceeding with subsequent experimental manipulations.

### Statistical analysis

The data are expressed as the mean ± standard deviation (SD) or mean ± standard error of the mean (SEM). Graphs were generated, and quantitative results were compared using Student’s *t* test. Significant differences between the groups were determined using one-way analysis of variance (ANOVA) followed by Tukey’s post hoc test. A *p* value < 0.05 was considered to indicate statistical significance. All of the statistical analyses were performed using Prism (GraphPad, La Jolla, CA, USA).

## Results

### FTH1 and FTL expression in pancreatic cancer

In our previous study, we found significantly greater SF levels in Taiwanese patients with pancreatic cancer than in healthy controls, and an additional pooled analysis of six case–control studies further confirmed the correlation between high SF and pancreatic cancer risk^14^. Recent studies have suggested that FTH1 and FTL are associated with cancer risk and that this risk may vary with cancer type; therefore, we hypothesized that differential FTH1 and/or FTL expression in ferritin is involved in pancreatic cancer risk^15,16^.

**Fig. 1:**
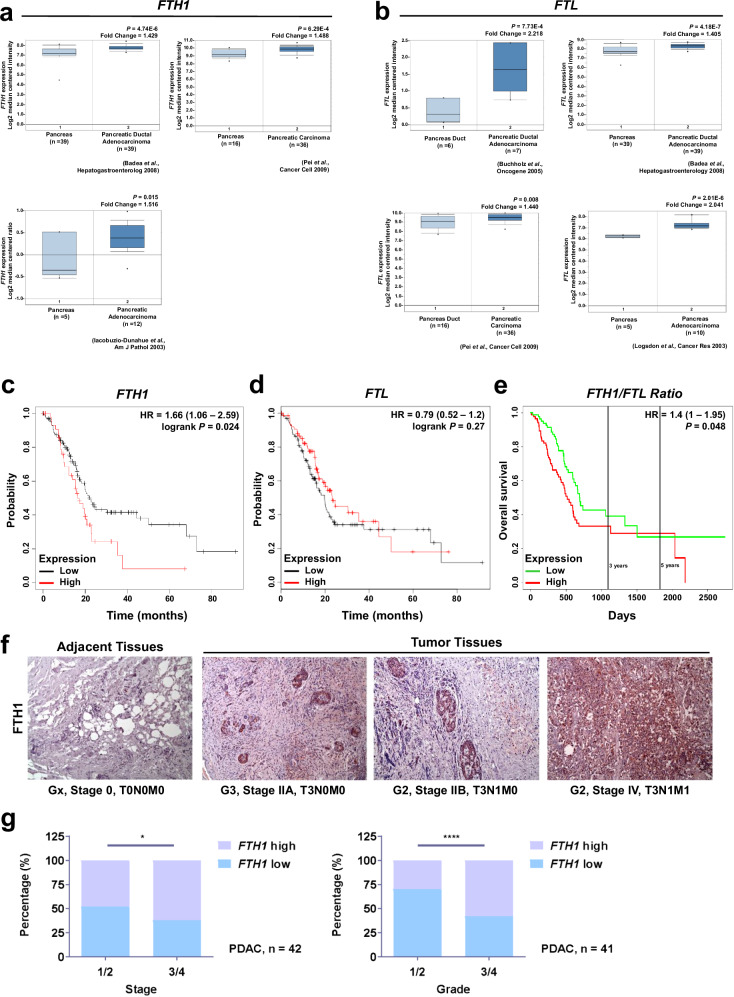
FTH1 and FTL expression in pancreatic cancer progression. **a**, **b** *FTH1* and *FTL* expression in pancreatic cancer. Data were retrieved from the Oncomine database (http://www.oncomine.org); *FTH1* and *FTL* mRNA levels were compared between normal pancreatic (left) and pancreatic cancer (right) tissues. **c**, **d** Survival curves for patients stratified by *FTH1* and *FTL* expression in pancreatic cancer by using Kaplan–Meier Plotter (www.kmplot.com). **e** Kaplan–Meier survival curve corrected for comparison of 170 patients with PDAC according to high versus low *FTH1*/*FTL* expression ratios from the TCGA dataset, assessed using PROGgeneV2 (http://genomics.jefferson.edu/proggene). **f** Representative IHC analysis depicting FTH1 expression in adjacent normal human tissue and pancreatic tumor tissues across various tumor grades and stages. Brown staining indicates FTH1 protein expression (magnification, ×100). *FTH1* expression is positively correlated with advanced-stage PDAC. **g** qRT‒PCR analysis of *FTH1* expression in pancreatic cancer tissues according to tumor stage (stage 1 and 2 vs. stage 3 and 4; left) and tumor grade (grade 1 and 2 vs. grade 3 and 4; right). The relative levels of *FTH1* expression are represented as ΔCP = CP of tested *FTH1* – CP of reference *FTH1*. The median ΔCP of patient samples was used as the cutoff to define high and low *FTH1* expression. HR hazard ratio; NT nontumor tissue; TNM tumor, node, and metastasis.

According to the Oncomine database data, both *FTH1* (Fig. 1a) and *FTL* (Fig. 1b) levels were elevated in pancreatic tumor tissues compared with those in normal pancreatic tissues; however, Kaplan–Meier survival curves demonstrated that high levels of *FTH1* (Fig. 1c) but not *FTL* (Fig. 1d) were associated with poor survival in patients with pancreatic cancer. To further confirm this finding, we generated PROGgeneV2 Kaplan–Meier survival curves by using data from The Cancer Genome Atlas (TCGA) and analyzed the association of the *FTH1*/*FTL* expression ratio with the overall survival of patients with pancreatic cancer (Fig. 1e). The results also confirmed that high *FTH1* expression is negatively correlated with the overall survival of patients with pancreatic cancer: patients with a higher *FTH1*/*FTL* expression ratio had significantly lower overall survival than did those with a lower *FTH1*/*FTL* expression ratio.

The results from the IHC staining analysis demonstrated that FTH1 expression was weak in normal pancreatic tissues but was considerably increased in malignant pancreatic tissues (Fig. 1f). Notably, FTH1 expression was strongly associated with the TNM stage, demonstrating that FTH1 is strongly expressed in human pancreatic tumor tissues and that its expression is positively correlated with a poor pancreatic cancer prognosis. Additional qPCR analysis also revealed that high *FTH1* mRNA levels were associated with advanced tumor stage and grade. The patients with PDAC were categorized into high (*FTH1* high) and low (*FTH1* low) expression groups with the median *FTH1* mRNA level as the cutoff; for instance, patients with advanced PDAC exhibited elevated *FTH1* mRNA levels (Fig. 1g). Approximately 60% of patients with advanced-stage and advanced-grade PDAC demonstrated high *FTH1* expression, whereas most patients with low-stage and low-grade PDAC demonstrated low *FTH1* expression.

### High FTH1 expression in pancreatic cancer is associated with *KRAS* mutation

**Fig. 2:**
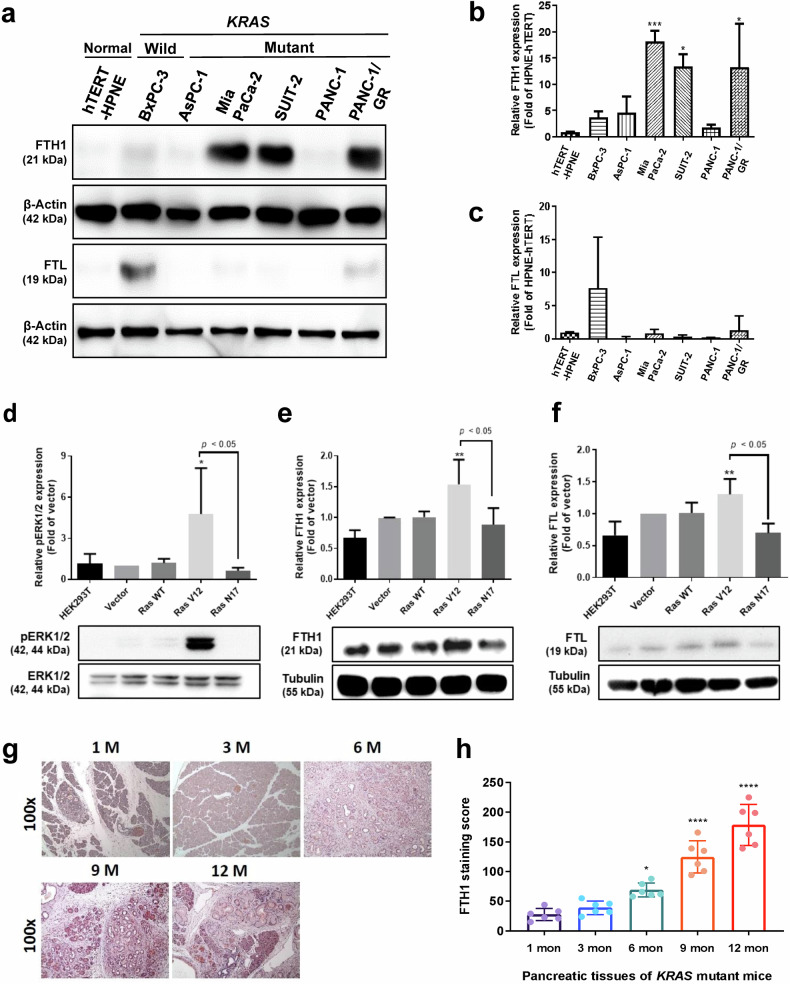
Mutant *KRAS* regulates FTH1 expression in pancreatic cancer. **a** Representative Western blot for FTH1 and FTL in hTERT-HPNE, BxPC-3, and *KRAS*-mutant pancreatic cancer (AsPC-1, Mia PaCa-2, SUIT-2, PANC-1, and PANC-1/GR) cells. β-Actin was used as a loading control. **b**, **c** show quantification of FTH1 and FTL expression in the indicated cells via ImageJ, normalized to β-actin, with bars indicating the mean fold change relative to hTERT-HPNE cell expression. The data are expressed as the means ± SDs (*n* = 3). *p < 0.05 and ***p < 0.001 compared with hTERT-HPNE cells. **d**–**f** HEK293T cells were transiently transfected with plasmids encoding vector, wild-type (WT), V12, or N17 plasmids and probed with **d** pERK1/2 and ERK1/2, **e** FTH1, and **f** FTL. Tubulin was used as a loading control. The data are expressed as the means ± SDs (*n* = 3). *p < 0.05 and **p < 0.01 compared with *RAS* V17 cells. **g** Representative images of FTH1 protein expression during pancreatic cancer development and progression in our KC mouse model. After tamoxifen administration, KC mice developed acinar-to-ductal metaplasia and PanIN. The mice were sacrificed during the indicated months, and their tissues were immunohistochemically stained for FTH1 (magnification, ×100). **h** IHC staining score for FTH1. The data are expressed as the means ± SDs (*n* = 6). *p < 0.05 and ****p < 0.0001.

To explore FTH1 and FTL expression in pancreatic cancer cells and their associations with *KRAS* mutation status, we analyzed FTH1 and FTL protein levels in hTERT-HPNE, *KRAS*-WT pancreatic cancer (BxPC-3), and *KRAS*-mutant pancreatic cancer (AsPC-1, Mia PaCa-2, SUIT-2, PANC-1, and PANC-1/GR) cells through Western blotting (Fig. 2a). The results revealed that hTERT-HPNE cells had relatively low FTH1 expression, whereas the FTH1 levels in the *KRAS*-mutant pancreatic cancer cell lines Mia PaCa-2 and SUIT-2 were approximately 18- and 13-fold higher, respectively, than those in hTERT-HPNE cells (Fig. 2b). Gemcitabine-resistant PANC-1/GR cells also exhibited considerably increased FTH1 expression, approximately 13-fold higher than that in hTERT-HPNE cells and 6.5-fold higher than that in PANC-1 cells. However, FTL expression did not significantly differ among the pancreatic cancer cell lines (Fig. 2c). However, most of the *KRAS*-mutant pancreatic cancer cell lines demonstrated lower FTL levels than did hTERT-HPNE cells. Compared with hTERT-HPNE cells, only *KRAS*-WT BxPC-3 and PANC-1/GR cells demonstrated a trend toward increased FTL expression, but this result was not significant.

To further confirm the association between FTH1 expression and *KRAS* mutation, we generated HEK293T cells with constitutively active *RAS* (V12). The HEK293T cell line was selected because it is regarded as a reliable host for transfection and is widely used to determine the mechanism mediated by *RAS* signaling^17–19^. The results indicated that in transfected *RAS* V12 cells, phosphorylated ERK1/2 (pERK1/2) was activated (Fig. 2d). Moreover, these cells had significantly higher FTH1 (Fig. 2e) and FTL (Fig. 2f) expression than *RAS* N17 and HEK293T cells.

We then assessed FTH1 expression in *LSL-Kras*^*G12D*^*/Pdx1*^*cre*^ (KC) mice through IHC staining. The KC mouse model, which closely replicates spontaneous PDAC progression in humans, undergoes acinar-to-ductal metaplasia and develops PanIN lesions within 3 to 6 months postpartum, progressing to PDAC pancreatic lesions by 6 months of age. In line with this disease progression, our study revealed an increase in FTH1 protein expression over time, as shown in Fig. 2g. This finding suggested a positive correlation between FTH1 levels and the progression of PDAC. The quantitative analysis presented in Fig. 2h revealed a progressive increase in FTH1 expression aligned with the development of PDAC in KC mice. Notably, the average scores for FTH1 immunostaining were markedly higher at the 6-, 9-, and 12-month intervals than at the 1-month benchmark, indicating a significant, time-dependent increase in FTH1 levels throughout PDAC progression.

### FTH1 participates in *KRAS*-mutant-mediated pancreatic cancer cell growth

**Fig. 3:**
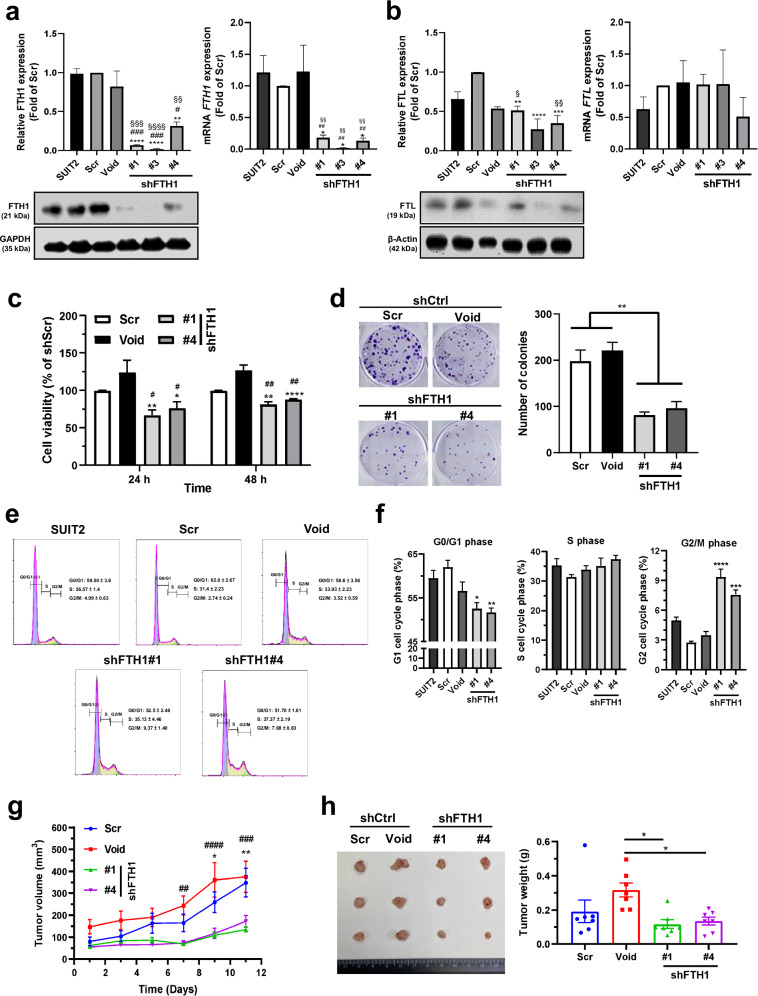
*FTH1* knockdown reduces *KRAS*-mutant pancreatic cancer cell viability and tumor growth. **a**, **b** FTH1 was knocked down in *KRAS*-mutant SUIT-2 cells through stable expression of shRNAs against FTH1 via the lentiviral expression constructs shCtrl (Scr, Void) and shFTH1 (#1, #3, and #4). Western blot (left) and qRT‒PCR (right) analyses of FTH1 and FTL after shFTH1 plasmid transfection into SUIT-2 cells. GAPDH or β-actin was used as a loading control. *p < 0.05, **p < 0.01, ***p < 0.001, and ****p < 0.0001 compared with Scr; ^#^*p* < 0.05, ^##^*p* < 0.01, and ^###^*p* < 0.001 com*p*ared with Void; and ^§^*p* *<* 0.05, ^§§^*p* < 0.01, ^§§§^*p* ^<^ 0.001, and ^§§§§^*p* < 0.0001 compared with SUIT-2 cells. **c** Cell viability in each cell group was determined using an MTT assay after 24 and 48 h. *p < 0.05 and **p < 0.01 compared with the Scr group; ^#^*p* < 0.05 and ^##^*p* < 0.01 compared with the Void group. **d** Each grou*p* of cells was plated in tri*p*licate in 6-well plates at 200 cells per well. After 8 days, colonies were counted using ImageJ after staining with 0.5% crystal violet in methanol. **p < 0.01 compared with the control (Scr and Void) group. **e** Cell cycle analysis through PI staining following flow cytometry of the transfected shCtrl (Scr or Void)-infected or shFTH1-infected SUIT-2 cells (#1 and #4). **f** G_0_/G_1_, S, and G_2_/M phase percentages of the indicated SUIT-2 cells were determined using FlowJo with the Dean–Jett–Fox model (with sync.peak). *p < 0.05, **p < 0.01, ***p < 0.0_0_1, and ****p < 0.0001 compared with the Scr group. The data are expressed as the means ± SEMs from three independent experiments (*n* ≥ 3). **g**, **h** Male NOD/SCID immunodeficient mice were subcutaneously injected in the back with tumor cells (Scr, Void, #1, and #4). **g** Tumor sizes were measured at various time points. *p < 0.05 and **p < 0.01 compared with the Scr group; ^##^*p* < 0.01, ^###^*p* < 0.001, and ^####^*p* < 0.0001 compared with the Void group. **h** Tumor sizes (left) and weights (right) in each group are shown. The data are expressed as the means ± SEMs (n = 7). ^#^*p* < 0.05 and ^##^*p* < 0.01 compared with the Void group.

To understand the role and function of FTH1 in PDAC, we knocked down FTH1 expression in *KRAS*-mutant SUIT-2 cells through lentiviral transduction. Here, the successful establishment of stable FTH1-knockdown cells (shFTH1#1, #3, and #4) was confirmed through Western blotting and qRT‒PCR; FTH1 expression was significantly lower in these cells than in controls (Scr and Void; Fig. 3a). Notably, the protein levels of FTL (Fig. 3b) in the SUIT-2/shFTH1#3 cells were significantly altered after shFTH1 viral infection compared with those in either the SUIT-2 or the Scr cells; therefore, we specifically selected the sh#1 and sh#4 FTH1-knockdown clones for further analyses. However, *FTL* mRNA expression did not significantly change.

We next examined the effects of *FTH1* on human PDAC cell viability by using an MTT assay (Fig. 3c). The results revealed that SUIT-2 cell viability was significantly decreased with *FTH1* knockdown compared with both the Scr and Void controls. A clonogenic assay was used to determine the long-term effects of FTH1 on pancreatic cancer cell proliferation and the survival of individual cells until they grew into colonies. Consistent with the MTT assay results, SUIT-2 cell colony growth was significantly decreased by approximately 45% and 50% after *FTH1* knockdown compared with that of the Scr and Void controls, respectively (Fig. 3d). We performed flow cytometry to determine whether FTH1 knockdown affects the cell cycle distribution of SUIT-2 cells. Compared with Scr, FTH1 knockdown reduced the percentage of SUIT-2 cells in the G_0_/G_1_ phase but increased the percentage of SUIT-2 cells in the G_2_/M phase (Fig. 3e, f).

To investigate the role of FTH1 in pancreatic cancer cell growth in vivo, SUIT-2 cells stably transfected with control shRNA (Scr and Void) or shFTH1 shRNA (shFTH1#1 and #4) were subcutaneously injected into the posterior flank of NOD/SCID male mice s.c. The results indicated that the shCtrl group demonstrated rapid tumor growth, with the tumor size increasing to 100–150 mm^3^ within 1 week; thus, the tumor volume of each group was measured every 2 days starting 1 week after the injection (Fig. 3g). Consistent with the in vitro results, FTH1 knockdown in SUIT-2 cells suppressed tumor growth: shFTH1 mice demonstrated significantly slower tumor growth on days 9 and 11 than did Scr mice and on days 7, 9, and 11 than did Void mice. Mice were sacrificed 12 days after tumor measurement; the tumors were then excised and weighed (Fig. 3h). The weights of the SUIT-2 tumors significantly decreased after FTH1 knockdown (*p* = 0.0140 and 0.0275 compared with sh#1 and sh#4, respectively). No significant reduction in tumor growth was observed in the shFTH1 groups compared with the Scr groups; nevertheless, relatively strong trends were noted.

**Fig. 4:**
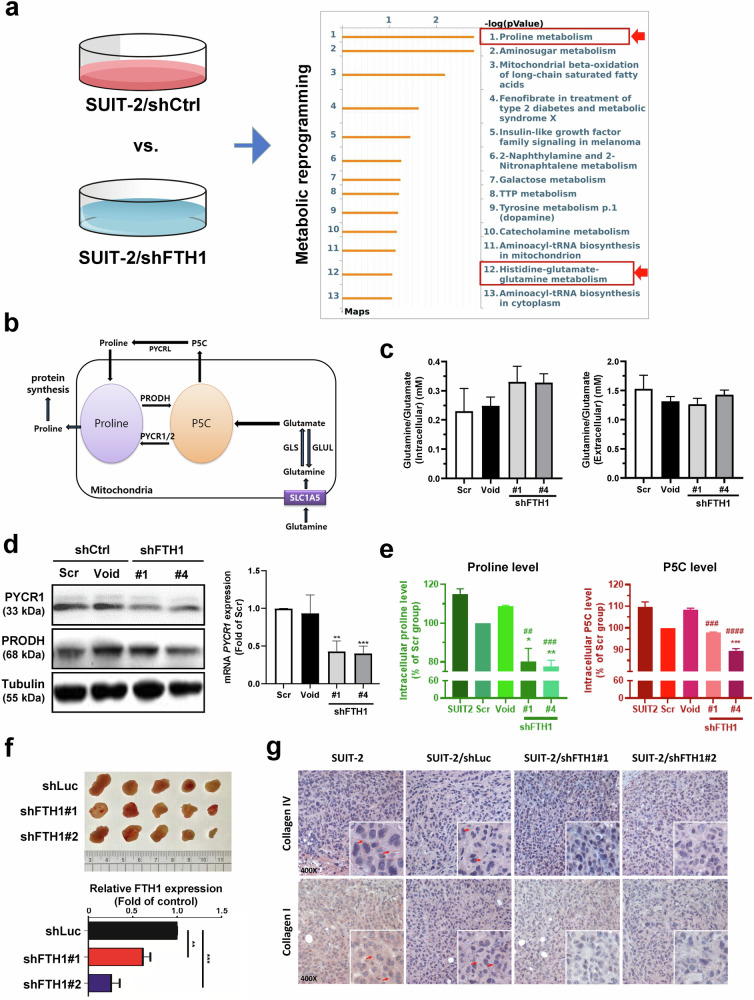
FTH1 mediates proline metabolic reprogramming in SUIT-2 cells. **a** Pathway analysis was conducted on metabolites differentially expressed in FTH1-knockdown SUIT-2 cells using metabolomics to identify significant pathways related to genes altered between shFTH1- and shCtrl-infected cells via the MASS Spectrum Browser. Proline metabolism has emerged as a key pathway involved in FTH1 regulation in pancreatic cancer cells. **b** Schematic of proline metabolism. **c** Glutamine/glutamate concentration ratios in the indicated SUIT-2 cells with shFTH1 knockdown. **d** Representative Western blots (left) of PYCR1 and PRODH and qRT‒PCR analysis (right) of PYCR1 expression in the indicated SUIT-2 cells with shFTH1 knockdown. Western blots were normalized to tubulin, with bars representing the mean fold change relative to Scr cells, while GAPDH served as the qRT‒PCR reference. **p < 0.01 and ***p < 0.001 compared with the Scr group. **e** Proline and P5C lev**e**ls in the indicated SUIT-2 cells with shFTH1 knockdown. Bars indicate the percentage change compared to the Scr control group. *p < 0.05, **p < 0.01, ***p < 0.001 compared with the Scr group; ^##^*p* < 0.01, ^###^*p* < 0.001, ^####^*p* < 0.0001 compared with the Void group. **f** Five male NOD/SCID mice were subcutaneously injected in the back with tumor cells (shLuc, shFTH1#1, and shFTH1#2). Tumor sizes (upper) and FTH1 expression (lower) in each group were reduced following FTH1 knockdown (*n* = 5), with shLuc serving as the control. **p < 0.01 and ***p < 0.001 compared with the shLuc group. **g** IHC staining revealed collagen I and IV in representative tumor sections from mice bearing subcutaneous tumors generated from control or FTH1-knockdown SUIT-2 cells at ×200 magnification.

To further confirm the role of FTH1 in *KRAS*-mutant-mediated pancreatic cancer cell growth, we assessed whether FTH1 overexpression can restore the effect of FTH1 knockdown on cell viability. MTT assay results demonstrated that the decrease in cell viability caused by FTH1 knockdown was significantly reversed by FTH1 overexpression after both 24 and 48 h (Supplementary Fig. 1a). Furthermore, FTH1 knockdown-mediated suppression of colony formation was reversed by FTH1 overexpression; however, significant differences were only detected between SUIT-2 cells infected with shFTH1#1 or ov-shFTH1#1 (Supplementary Fig. 1b). As shown in Fig. 4c, the cell cycle analysis results indicated that FTH1 may be crucial for cell cycle regulation in pancreatic cancer cells: with FTH1 expression rescue, the percentages of cells in the G_0_/G1 phase increased from 45.2% to 54.1% in SUIT-2/shFTH1#1 cells and from 44.6% to 52.0% in SUIT-2/shFTH1#4 cells, followed by a substantial reduction in the percentage of cells in the G2/M phase (Supplementary Fig. 1c, d).

We also confirmed the rescue effect of FTH1 in vivo: FTH1 knockdown in SUIT-2 cells suppressed tumor growth, whereas the restoration of FTH1 expression rescued its tumor-suppressive effect. The SUIT-2 xenograft tumor volume was significantly lower with FTH1 knockdown (284 ± 47 mm^3^) than without FTH1 knockdown (970 ± 119 mm^3^ for SUIT-2 cells and 1007 ± 129 mm^3^ for the Scr group) and with rescued FTH1 expression (431 ± 44 mm^3^) (Supplementary Fig. 1e). Additionally, the mean tumor weight was lower in the FTH1 knockdown group (0.180 g) than in the control group (0.532 g for the SUIT-2 group and 0.604 g for the Scr group) and greater than that in the FTH1 rescue group (0.334 g) (Supplementary Fig. 1f).

### FTH1-mediated proline metabolism is involved in pancreatic cancer cell growth

Altered metabolism is a hallmark of cancer; thus, we further investigated FTH1-mediated metabolic reprogramming in pancreatic cancer^20,21^. We used LC-MS-based metabolomics to determine the changes in the metabolic profiles of FTH1-knockdown SUIT-2 cells and identify candidate metabolic pathways involved in FTH1-mediated regulation. Pathway enrichment analysis via metabolomics indicated that the proline cycle and glutamate-glutamine metabolism in SUIT-2 cells were markedly altered by *FTH1* knockdown (Fig. 4a and Supplementary Table 2). Proline metabolism is schematically presented in Fig. 4b.

*KRAS* alters glucose and glutamine utilization^22^. We previously reported that FTH1 is strongly expressed in many *KRAS*-mutant pancreatic cancer cells (Fig. 2a, b); thus, extracellular and intracellular glutamine and glutamate contents were measured to evaluate whether FTH1 is associated with a metabolic shift in glutamine metabolism (Fig. 4c). Compared with Scr and Void, FTH1 knockdown led to slight (and nonsignificant) increases in intracellular glutamine and glutamate levels. However, the extracellular glutamine and glutamate contents did not differ between the control and FTH1-knockdown SUIT-2 cells.

To further explore how FTH1 is linked to the reprogramming of proline metabolism in pancreatic cancer cells, we measured the protein expression of proline metabolism-associated molecules, PYCR1 and PRODH, in control and shFTH1-infected SUIT-2 cells through Western blotting (Fig. 4d, left panel). Compared with that in the shCtrl group, a significant reduction in the protein expression of PYCR1, but not PRODH, was observed following FTH1 knockdown in comparison to the shCtrl group. Similarly, *PYCR1* mRNA levels were significantly decreased after FTH1 suppression (Fig. 4d, right panel). Furthermore, after FTH1 knockdown, there were significant decreases in the concentrations of proline and P5C (Fig. 4e). We analyzed whether *KRAS* mutation status was correlated with PYCR1 and PRODH protein expression; however, the differences in PYCR1 and PRODH expression between *RAS* V12 cells and *RAS* N17 and HEK293T cells were not significant (Supplementary Fig. 2).

Because high collagen content is found in pancreatic cancer and collagen-derived proline plays an oncogenic role in promoting PDAC survival^23^, we wondered whether FTH1 also participates in collagen matrix production and consequently contributes to pancreatic cancer progression. shLuc- and shFTH1-infected SUIT-2 cells were injected into the posterior flank of NOD/SCID mice s.c.; next, their tumor weights were measured and their collagen I and IV protein expression were evaluated by IHC. We confirmed the successful establishment of stable FTH1-knockdown cells (Supplementary Fig. 3) and the tumor-suppressive effects of FTH1 in SUIT-2 cells (Fig. 4f). Intense collagen I and IV staining was observed in shLuc-infected and noninfected SUIT-2 cells; the staining intensity was reduced after FTH1 knockdown (Fig. 4g).

### FTH1–PYCR1 crosstalk mediates pancreatic cancer progression

**Fig. 5:**
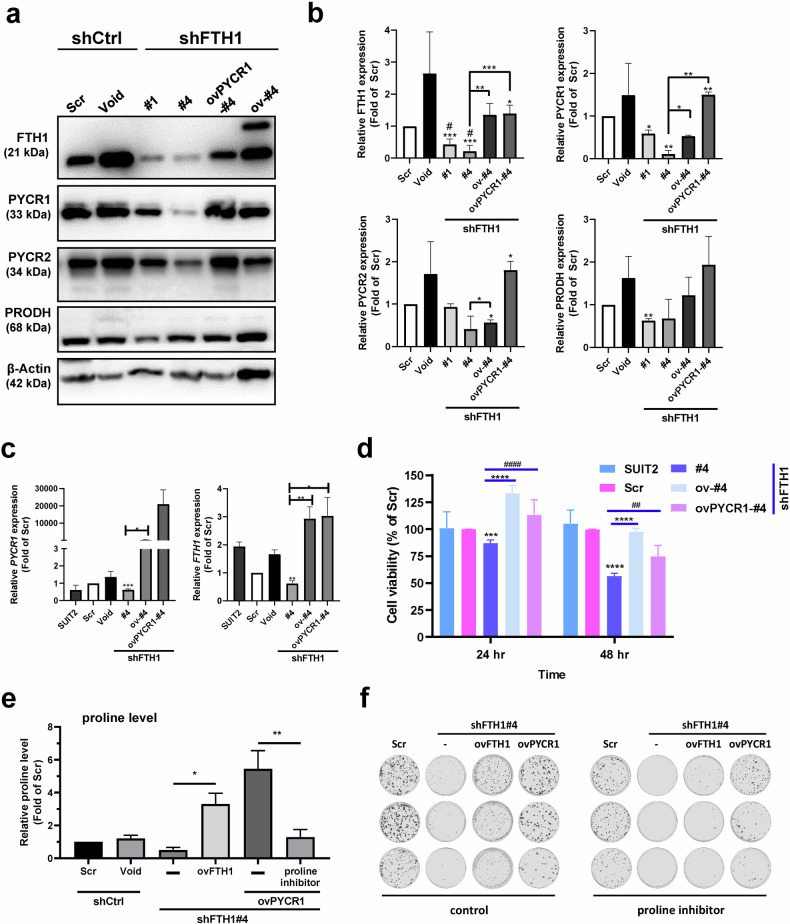
FTH1 interacts with PYCR1 to regulate proline metabolism, which contributes to pancreatic cancer cell viability. **a** FTH1, PYCR1, PYCR2, and PRODH expression in shCtrl-infected (Scr), shFTH1-infected (#1 and #4) SUIT-2 cells, and shFTH1-rescued FTH1 (ov-#4) cells and PYCR1 overexpression in SUIT-2/shFTH1#4 (ovPYCR1-#4) cells were examined through Western blotting. **b** Western blots were normalized to β-actin, and each bar shows the mean fold change relative to expression in Scr and Void cells. The data are expressed as the means ± SEMs from at least two independent experiments. *p < 0.05, **p < 0.01, ***p < 0.001 compared with the Scr group and ^#^*p* < 0.05 compared with the Void group. **c** *PYCR1* (left) and *FTH1* (right) mRNA expression in the indicated cells was analyzed using qRT‒PCR. *GAPDH* was used as a loading control. The data are expressed as the means ± SEMs from three independent experiments (*n* = 3). *p < 0.05, **p < 0.01, ***p < 0.001 compared with the Scr group. **d** Cell viability of each group of cells was determined using the MTT assay at 24 and 48 h. The data are expressed as the means ± SEMs from three independent experiments (*n* = 3). ***p < 0.001 and ****p < 0.0001 compared with either the Scr or rescued shFTH1 group and ^##^*p* < 0.01 and ^####^*p* < 0.0001 compared with the #4 and ovPYCR1-#4 groups. **e** Proline levels in the designated SUIT-2 cells were measured after exposure to a proline inhibitor (2 mM for 48 hr). Bars indicate the fold change compared to the Scr control group. *p < 0.05, **p < 0.01. **f** The indicated proline inhibitor-treated SUIT-2 cells were seeded at 200 per well in 6-well plates and incubated with a *p*roline inhibitor for 8 days. After incubation, the colonies were stained with 0.5% crystal violet in methanol.

We subsequently performed rescue experiments on FTH1-overexpressing shFTH1-infected SUIT-2 cells to further confirm the role of FTH1 in proline metabolism dysregulation and pancreatic cancer progression. Western blot analysis revealed a marked decrease in the PYCR1 protein level following FTH1 knockdown in SUIT-2 cells, while the PRODH level remained unchanged (Fig. 5a). The quantitative data corroborated this observation, revealing a notable downregulation of FTH1 and subsequent reduction in PYCR1 expression, particularly in the shFTH1#4 clone. Interestingly, the overexpression of PYCR1 in clone #4 rescued FTH1 expression, and conversely, the overexpression of FTH1 rescued PYCR1 expression in these cells, suggesting a positive feedback loop between FTH1 and PYCR1 (Fig. 5b). However, this regulatory mechanism does not extend to PRODH, as its protein levels remained largely unaffected by either FTH1 or PYCR1 overexpression. This distinction highlights a specific interplay between FTH1 and PYCR1 that does not involve PRODH. As shown in Fig. 5c, a significant decrease in the PYCR1 mRNA level upon FTH1 knockdown further supports the posttranscriptional regulation of PYCR1 by FTH1. Cell viability assays revealed that suppression of FTH1 resulted in a significant decrease in the viability of SUIT-2 cells, while overexpression of either FTH1 or PYCR1 partially rescued this effect (Fig. 5d), highlighting the role of FTH1–PYCR1 crosstalk in cellular survival. Moreover, Fig. 5e shows a substantial increase in the levels of proline following FTH1 expression. The further decrease in proline levels upon treatment with a proline inhibitor suggested that proline itself may modulate the effects of FTH1, creating a feedback loop that impacts cell metabolism and survival. The clonogenic capacity for proline suppression was significantly compromised in cells with FTH1 and PYCR1 expression (Fig. 5f), suggesting that FTH1/PYCR1-mediated proline positively regulates the proliferative potential of these cells. This finding is consistent with the notion that the FTH1–PYCR1 axis plays a crucial role in the regulation of proline metabolism and pancreatic cancer progression. Collectively, these results suggest a complex regulatory network in which FTH1 influences pancreatic cancer progression by modulating PYCR1 expression, which in turn may be part of a feedback loop involving proline that controls the effects of FTH1, thus impacting cell viability and proliferation.

**Fig. 6:**
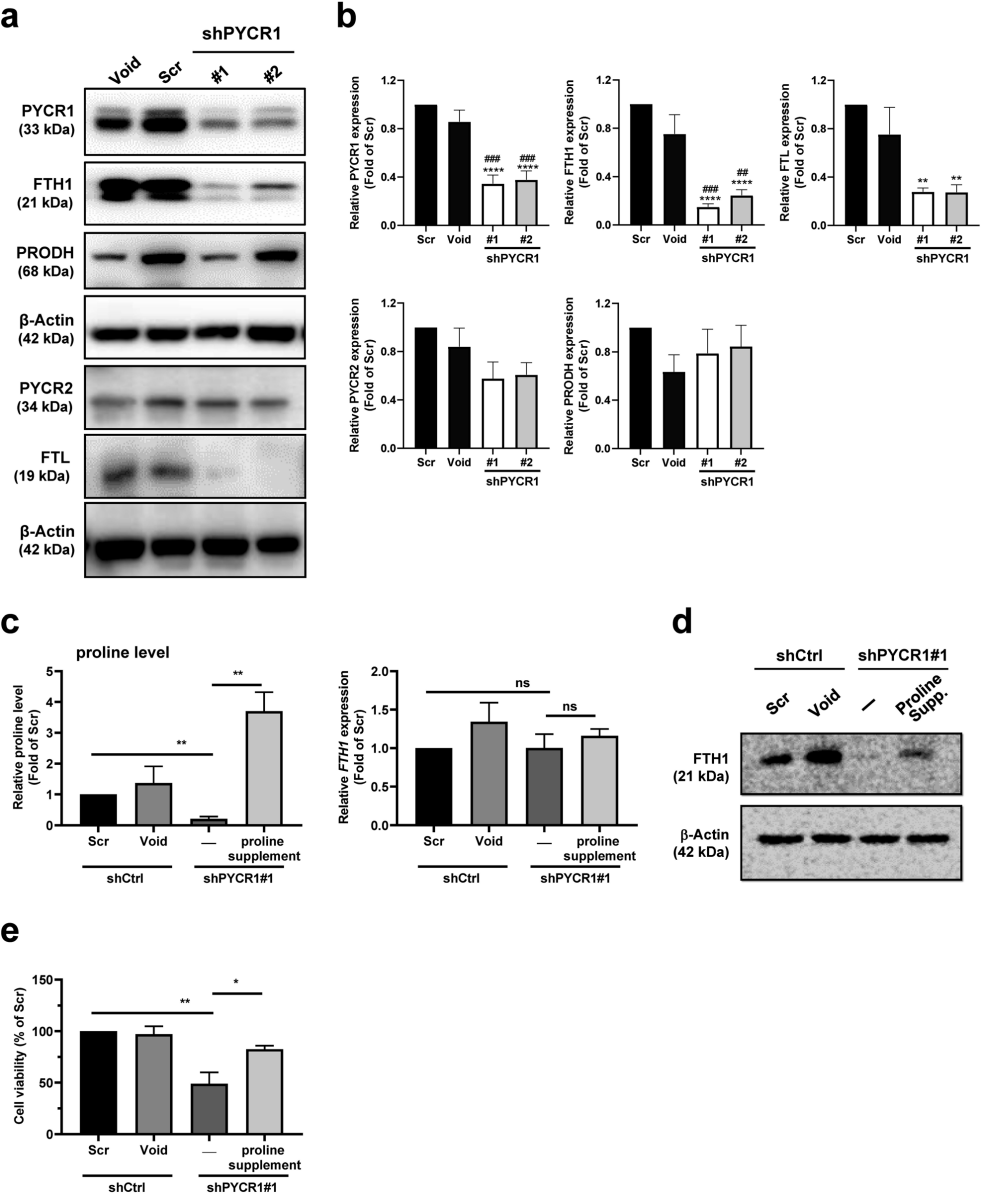
Proline supplementation reversed changes in FTH1 protein expression and cell viability in PYCR1-knockdown cells. **a** PYCR1 was knocked down through stable expression of shRNAs against PYCR1 from the following lentiviral expression constructs: Scr, Void (control), and shPYCR1#1 and shPYCR1#2. **b** Quantification of PYCR1, PYCR2, PRODH, FTH1, and FTL expression in the indicated cells was performed via ImageJ. Blots were normalized to β-actin, with bars indicating the mean fold change relative to Scr cell expression. The data are shown as the means ± SEMs; **p < 0.01, ****p < 0.0001 vs. the Scr group; ##*p* < 0.01 vs. the Void group. **c** Effects of PYCR1 knockdown and proline supplementation on proline levels and *FTH1* mRNA expression. Left panel: Proline levels relative to those in the Scr control group in shCtrl, shPYCR1#1, and 200 μM proline-supplemented shPYCR1#1 cells. Right panel: *FTH1* mRNA expression relative to that in the Scr control group under the same conditions. Bars represent the mean ± SEM; **p < 0.01 indicates significance, while ns denotes not significant. **d** Western blot analysis showing the protein expression of FTH1. The expression was compared across SUIT-2 cells with control shRNA (Scr and Void), without treatment (−), and cells with 200 μM proline supplementation (Proline Supp.) following shPYCR1#1 knockdown. β-Actin served as a loading control. **e** MTT assay-based cell viability assessment in SUIT-2 cells following shRNA-mediated knockdown and 48-hour proline supplementation. Viability percentages are relative to those of the Scr control group, indicating the effects of scrambled shRNA (Scr), vector control (Void), shRNA control (shCtrl), and PYCR1 knockdown (shPYCR1#1) with or without the addition of 200 μM proline for 48 hr. The data are shown as the mean ± SEM; *p < 0.05, **p < 0.01.

To elucidate the complex interplay between FTH1 and PYCR1 in pancreatic cancer cells, Western blot analyses were performed. These analyses revealed a marked decrease in PYCR1 protein expression in clones #1 and #2, indicating effective knockdown. This reduction was specific to PYCR1, as the protein levels of PRODH and PYCR2 remained unchanged. Intriguingly, the loss of PYCR1 resulted in a reduction in FTH1 protein levels compared to both Scr and Void controls (Fig. 6a), further emphasizing the reciprocal crosstalk between FTH1 and PYCR1. The specificity of PYCR1 knockdown and the resulting regulatory effects on FTH1 expression were quantitatively validated by Western blot analysis (Fig. 6b). Further examination of proline levels revealed a significant decrease in cells with reduced PYCR1, which could be reversed by proline supplementation, underscoring the role of PYCR1 in proline biosynthesis (Fig. 6c). Additionally, Western blot analysis demonstrated that proline supplementation led to upregulation of the FTH1 protein in PYCR1-knockdown cells (Fig. 6d), suggesting that proline availability significantly influences FTH1 protein stability. Moreover, cell viability assays revealed a notable decrease in the survival of PYCR1 knockdown cells, which was ameliorated upon the addition of proline, underscoring the vital role of proline in cell viability (Fig. 6e). These observations collectively indicate that PYCR1 serves as a principal regulator of proline biosynthesis and that proline plays a role in the compensatory upregulation of FTH1, suggesting an adaptive mechanism in pancreatic cancer cells.

Based on these findings, the presence of FTH1–PYCR1 crosstalk was investigated in other PDAC cell lines. Mia PaCa-2 cells infected with shFTH1 exhibited significant decreases in cell viability, clonogenic potential, and PYCR1 protein levels, reinforcing the existence of this interaction in the modulation of PDAC cell progression (Supplementary Fig. 4a–d). Additionally, the assessment of proline and P5C levels in shFTH1-infected SUIT-2 and Mia PaCa-2 cells indicated that FTH1 knockdown led to a reduction in intracellular proline levels in both cell lines, while P5C levels decreased only in SUIT-2 cells following FTH1 suppression (Supplementary Fig. 4e). Taken together, these findings highlight the critical regulatory axis of FTH1 and PYCR1 in pancreatic cancer cell metabolism and survival, suggesting a shared pathway that influences malignancy and potential treatment targets in PDAC.

### FTH1 regulates PYCR1 expression via miRNA modulation

**Fig. 7:**
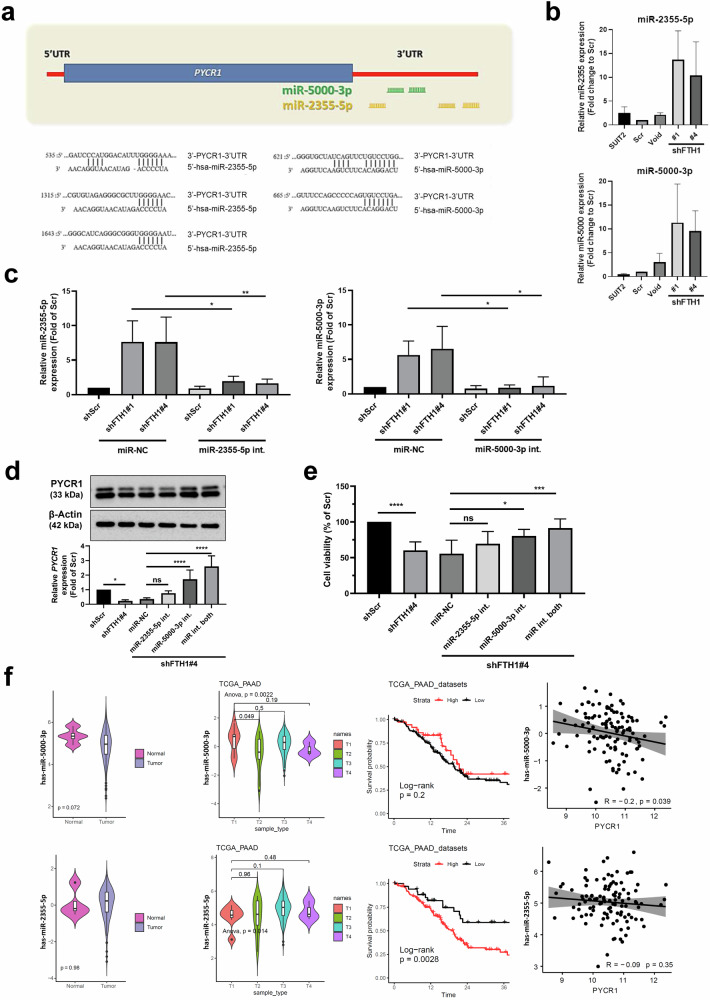
FTH1 knockdown suppresses PYCR1 expression via miRNA regulation. **a** Target prediction via TargetScan revealed that the 3′-UTR sequence of *PYCR1* contains putative binding sites for *miR-2355-5p* and *miR-5000-3p*. **b** Expression of *miR-2355-5p* and *miR-5000-3p* in the indicated SUIT-2, transfected shCtrl (Scr), and shFTH1 knockdown SUIT-2 (#1 and #4) cells was analyzed using qRT‒PCR. The qRT‒PCR data were normalized to *U47* levels in each individual sample, and the bar plot shows the fold changes in Scr expression. The data are expressed as the means ± SEMs from three independent experiments (*n* = 3). **c** Differential miRNA expression upon FTH1 knockdown in SUIT-2 cells. Left panel: Relative expression of miR-2355-5p in shScr, shFTH1#1, and shFTH1#4 cells treated with either miR-NC (negative control) or the miR-2355-5p inhibitor (int.). Right panel: Relative expression of miR-5000-3p under the same conditions. The expression was normalized to that in the shScr group, with the bars representing the mean ± SEM. Significance is denoted by asterisks (*p < 0.05, **p < 0.01). **d** Impact of FTH1 knock**d**own and miRNA inhibition on PYCR1 protein (upper panel) and mRNA expression (bottom panel) in SUIT-2 cells. β-Actin was used as a loading control for the WBs. Relative *PYCR1* mRNA expression in shScr, shFTH1#4, and miR-NC cells and in cells treated with miR-2355-5p or miR-5000-3p inhibitors, both individually and in combination (int. both), normalized to that in shScr-treated cells. **e** Cell viability of the same groups, expressed as a percentage of the shScr control. Significance is indicated as *p < 0.05, ***p < 0.001, and ****p < 0.0001. **f** Multipanel analysis of miRNA expression and correlation with survival and PYCR1 levels in PAAD. Top left: Violin plots comparing the expression levels of hsa-miR-5000-3p in normal and tumor tissues, *p* = 0.072. Top second from left: Violin plots of hsa-miR-5000-3p expression across different tumor stages (T1-T4), with ANOVA *p* = 0.0022. Top middle: Kaplan‒Meier survival curves stratified by high and low expression of hsa-miR-5000-3p, log-rank *p* = 0.2. Top right: Scatter plot depicting the inverse correlation between hsa-miR-5000-3p and PYCR1 expression, *R* = −0.2, *p* = 0.039. Bottom left: Violin plots showing the expression levels of hsa-miR-2355-5p in normal and tumor tissues, *p* = 0.98. Bottom second from left: Violin plots of hsa-miR-2355-5p expression across different tumor stages; ANOVA, *p* = 0.014. Bottom middle: Kaplan‒Meier curves based on hsa-miR-2355-5p expression; log-rank *p* = 0.0028. Bottom right: Scatter plot showing no significant correlation between hsa-miR-2355-5p and PYCR1 expression, *R* = −0.09, *p* = 0.35. The data were derived from the TCGA_PAAD dataset.

A study suggested that *miR-2355-5p*, *miR-3150a-3p*, and *miR-5000-3p* are candidate miRNAs that regulate *PYCR1* expression in hepatocellular carcinoma; additional qRT‒PCR results indicated that *miR-2355-5p* may be an upstream regulator of *PYCR1* mRNA^24^. Because we found that *PYCR1* mRNA levels are controlled by *FTH1*, we explored the expression profiles of candidate miRNAs possibly associated with FTH1 dysregulation in SUIT-2 cells. To confirm whether *miR-2355-5p* and *miR-5000-3p* target *PYCR1*, target prediction was performed using TargetScan; we observed that the 3′-UTR sequence of *PYCR1* contains putative binding sites for *miR-2355-5p* and *miR-5000-3p* (Fig. 7a). We then used qRT‒PCR to measure *miR-2355-5p* and *miR-5000-3p* expression in control and shFTH1-infected SUIT-2 cells, and the results suggested that the *PYCR1* level was suppressed in FTH1-knockdown SUIT-2 cells through *miR-2355-5p* and *miR-5000-3p* upregulation (Fig. 7b). We introduced inhibitors targeting miR-2355-5p and miR-5000-3p into SUIT-2/shFTH1 cells (Fig. 7c), and subsequent analysis of PYCR1 expression revealed that inhibiting miR-5000-3p mitigated the suppression of *PYCR1* expression (Fig. 7d). This outcome indicates that miR-5000-3p contributes to the downregulation of *PYCR1*, with the inhibition of PYCR1 leading to the restoration of PYCR1 levels and a significant increase in the survival of SUIT-2/shFTH1 cells when miR-5000-3p was inhibited, as shown in Fig. 7e. Furthermore, expression analysis of miR-2355-5p and miR-5000-3p in pancreatic cancer patient samples from the TCGA database demonstrated an inverse correlation between miR-5000-3p and *PYCR1* expression (Fig. 7f). Spearman’s rank correlation tests were utilized to determine the associations among patients harboring *KRAS* mutations, revealing a negative correlation for miR-5000-3p but not for miR-2355-5p. Kaplan–Meier survival plots suggested a nonsignificant trend toward better survival outcomes with higher miR-5000-3p expression. These results collectively suggest that miR-5000-3p is a potential modulator of *PYCR1* expression, influencing FTH1-mediated pancreatic cancer progression and possibly patient survival.

### DFX treatment significantly reduces FTH1-knockdown SUIT-2 cell viability

**Fig. 8:**
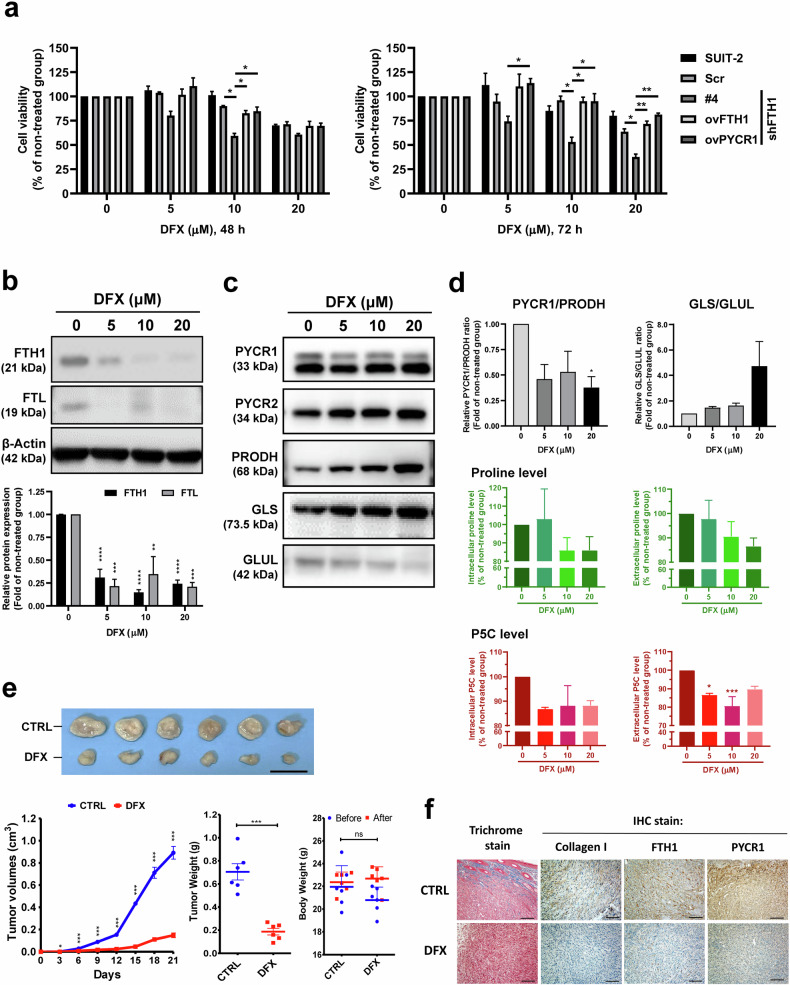
DFX treatment significantly reduces FTH1-mediated tumor growth. **a** The effect of deferasirox (DFX) on the viability of SUIT-2 cells and various genetically modified cell lines. The left graph displays cell viability after 48 h of treatment with 0, 5, 10, or 20 µM DFX, while the right graph shows the results after 72 h of treatment. The following cell lines were used: parental SUIT-2, scrambled control (Scr), shFTH1 clone #4 (shFTH1#4), and cells overexpressing FTH1 (ovFTH1) and PYCR1 (ovPYCR1). Viability is presented as a percentage of the untreated control group for each cell line, with bars denoting the mean ± SEM. Significance is indicated by *p < 0.05 and **p < 0.01. **b** The immunoblots at the top display bands for FTH1 and FTL after 48 hr of treatment with 0, 5, 10, or 20 µM DFX. β-Actin served as the loading control. The bar graph below shows the quantified protein expression normalized to that in the untreated group, with black bars representing FTH1 and gray bars representing FTL. The data are presented as the mean ± SEM (***p < 0.001). **c** The blot displays bands for PYCR1, PYCR2, PRODH, GLUL, and GLS at DFX concentrations of 0, 5, 10, and 20 µM. β-Actin served as a loading control. **d** Top panels display the relative enzyme expression ratios of PYCR1/PRODH and GLS/GLUL, normalized to the untreated control. The bottom panels show the intracellular and extracellular levels of proline and P5C (pyrroline-5-carboxylate), which are presented as percentages of those in the nontreated group. All of the data are presented as the means ± SEMs after treatment with 0, 5, 10, or 20 µM DFX. *p < 0.05, ***p < 0.001. **e** Top, Re*p*resentative images of tumors excised from mice pretreated with PBS (CTRL) or DFX (160 mg/kg). Scale bars: 20 mm. Bottom left–tumor growth curves at 21 days postimplantation of 1 × 10^6^ DesPanc03 mouse pancreatic cancer cells, with DFX treatment continuing every three days. Bottom middle—Final tumor weight comparison indicating a significant reduction in the DFX group. Bottom right—No significant changes in body weight suggest minimal systemic toxicity of DFX. **f** Trichrome and IHC staining of tumor sections for collagen I, FTH1, and PYCR1 reflecting the microenvironmental alterations caused by DFX treatment. The data are shown as the mean ± SEM, with *p < 0.05, ***p < 0.001 indicating significance, and ns denoting nonsignificance. Scale bars: 100 µm.

Deferasirox (DFX) is a novel oral iron chelator, and studies have demonstrated its potential role as a new pancreatic cancer therapy^25–27^. In mice, oral DFX treatment has been demonstrated to significantly reduce the average tumor xenograft volume, and this effect may have involved decreased SF levels^25,26^. Next, we found that DFX exhibited enhanced inhibitory effects on FTH1-knockdown SUIT-2 cells, significantly reducing cell viability at varying concentrations after both 48 and 72 h (Supplementary Fig. 5a). For PANC-1/GR cells, DFX alone decreased viability, whereas for PANC-1 cells, a combination of DFX and gemcitabine had a similar effect. The combined treatment notably sensitized PANC-1/GR cells, leading to a marked reduction in cell viability (Supplementary Fig. 5b). We also observed that FTH1 and PYCR1 overexpression in SUIT-2/shFTH1#4 cells considerably rescued the inhibitory effects of 10 µM and 20 µM DFX after 72 h, suggesting that FTH1/PYCR1 expression is key for the anti–pancreatic cancer activity of DFX (Fig. 8a). To confirm the association between DFX treatment and FTH1 expression in pancreatic cancer cells, SUIT-2 cells were treated with various concentrations of DFX for 48 h, and lysates were collected for Western blotting. We found that the expression of FTH1 and FTL significantly decreased with DFX treatment (Fig. 8b) and that the PYCR1/PRODH expression ratio decreased with 20 μM DFX treatment (Fig. 8d, c), suggesting that a high dose of DFX treatment inhibits the crosstalk between FTH1 and PYCR1 and leads to the suppression of pancreatic cancer cell viability. There was a strong trend where the GLS/glutamate-ammonia ligase (GLUL) expression ratio increased with DFX treatment in a dose-dependent manner (Fig. 8d), but without any statistical significance. Proline and P5C levels were measured in DFX-treated cells, as shown in Fig. 8d, bottom panel. While the intracellular and extracellular proline levels tended to decrease with increasing DFX concentration, these changes were not statistically significant. Conversely, extracellular P5C levels significantly decreased with 5 and 10 μM DFX treatment, indicating a DFX-mediated alteration in the proline synthesis pathway. These results suggest that DFX treatment decreased the PYCR1/PRODH expression ratio at higher doses, suggesting the disruption of FTH1–PYCR1 crosstalk and subsequent suppression of cell viability.

Furthermore, the in vivo results revealed that the oral administration of DFX significantly reduced the tumor xenograft volume in mice (Fig. 8e), consistent with earlier findings on the efficacy of DFX in reducing serum ferritin (SF) levels and tumor growth. This finding corroborates the hypothesis that DFX could diminish pancreatic cancer progression. Finally, Fig. 8f provides histological evidence from trichrome and immunohistochemistry (IHC) staining. These stains show the effects of DFX on the tumor microenvironment, particularly regarding collagen deposition and the expression of FTH1 and PYCR1, which are key to understanding the mechanistic impact of DFX on pancreatic cancer cells and tissues. Taken together, these results suggest that DFX treatment significantly affects pancreatic cancer cell viability, potentially through the modulation of FTH1 expression and alterations in proline and P5C metabolism, suggesting a promising therapeutic avenue for targeting pancreatic cancer.

### Associations between proline biosynthesis enzyme expression and pancreatic cancer patient survival

The Kaplan–Meier Plotter database was utilized to determine the associations between the expression of proline biosynthesis enzymes (*PYCR1*, *PYCR2*, *PYCR3*, and *PRODH*) and overall survival of pancreatic cancer patients, as depicted in Supplementary Fig. 6a. Our analysis indicated that higher *PYCR1* expression was associated with poorer overall survival, mirroring our in vitro findings showing that PYCR1 overexpression in SUIT-2/shFTH1 cells not only compensated for FTH1 expression but also enhanced cell viability and colony formation capacity. Conversely, higher *PYCR2* expression correlated with better overall survival, suggesting that PYCR2 plays a divergent role from that of PYCR1. *PYCR3* expression did not appear to impact overall survival. Additionally, pancreatic cancer patients with elevated *PRODH* expression had a shorter median overall survival than those with lower PRODH expression, highlighting the potential prognostic value of these enzymes in pancreatic cancer.

Using PROGgene V2 and survival analysis with the GSE21501 dataset, we found that concurrent high expression of *FTH1* and *PYCR1* correlated with poorer survival in human pancreatic cancer patients, as shown in Supplementary Fig. 6b. Similarly, TCGA data analysis revealed that higher levels of *FTH1* and *PYCR1* were associated with worse patient outcomes. These clinical findings align with our in vitro evidence, suggesting that the FTH1–PYCR1 interaction enhances oncogenic activity in *KRAS*-mutant PDAC cells. Further analysis of TCGA data indicated that the co-occurrence of *FTH1* expression and *KRAS* mutation leads to a poorer prognosis, reflected by shorter overall survival and a higher hazard ratio, reaffirming the in vitro results.

## Discussion

The present study expanded on previous observations of the association between SF and pancreatic cancer risk, specifically exploring the molecular mechanism underlying the involvement of ferritin in pancreatic cancer progression^14^. Surgical removal of tumors can reduce SF levels by approximately 50%, suggesting that the elevation in SF may be due to localized ferritin release within the tumor site, and ferritin subunit expression ratios vary among different species and cell types^16,28,29^. In this study, FTH1 expression was upregulated in most *KRAS*-mutant human pancreatic cancer cells and clinical pancreatic cancer tissues, contributing to PDAC progression through positive crosstalk with PYCR1 and promoting proline metabolism dysregulation (Supplementary Fig. 7).

Cancer results from the accumulation of genetic alterations, including mutations in canonical oncogenes, DNA mismatch repair genes, and tumor suppressor genes^30^. PanIN-to-PDAC progression is also associated with the accumulation of gene mutations, such as *KRAS* mutations, in >90% of patients with PDAC^31^. Here, we found that FTH1 may be a promising therapeutic target for *KRAS-*mutant pancreatic cancer. In *KRAS*-mutant SUIT-2 cells, FTH1 knockdown led to significant decreases in cancer cell viability via G2/M cell cycle arrest in vitro as well as tumor growth suppression in the SUIT-2 xenograft model in vivo (Fig. 3). Notably, we found variations in ferritin subunit expression among the pancreatic cancer cell lines with respect to the *KRAS* mutation status. Another ferritin subunit type, FTL, exhibited low expression in *KRAS*-mutant cells but relatively high expression in the *KRAS*-WT pancreatic cancer cell line BxPC-3, indicating that variations in FTH1 and FTL expression may be key factors driving pancreatic cancer progression in patients with different *KRAS* mutation statuses. The current results are consistent with the online database data: strong *FTH1* (Fig. 1c) but not *FTL* (Fig. 1d) expression is significantly correlated with worsened survival of patients with pancreatic cancer as well as in those with high *FTH1*–*KRAS* co-occurrence (Supplementary Fig. 6b). Currently, selective delivery mechanisms, such as antibody‒drug conjugates (ADCs) and ligand-directed systems, present a viable option to deliver FTH1 inhibitors by recognizing tumor-specific markers, thereby sparing normal cells^32^. Additionally, the differential expression of microRNAs (miRNAs) that regulate FTH1 in cancer cells suggests the potential for designing cancer-specific miRNA mimics or antagomirs to precisely modulate FTH1 expression^33^. Empirical evidence from the use of iron chelators such as deferasirox in clinical settings suggests that targeting iron metabolism can be effective with a manageable safety profile, given the proper therapeutic window and dose optimization^34^. The evidence of safety and efficacy from clinical trials supports the feasibility of selectively targeting FTH1 in cancer therapy, opening up new avenues for treatment strategies for *KRAS*-mutant pancreatic cancer.

Recent literature has highlighted the pivotal role of altered metabolic pathways in supporting the survival and growth of *KRAS*-driven cancers^35–37^. Our metabolic profiling results indicated that FTH1 knockdown significantly disrupted proline metabolism in SUIT-2 cells. Proline metabolism involves the conversion of glutamate to P5C by ALDH18A1, followed by its subsequent conversion to proline via PYCR enzymes^38^. Among the PYCR isoforms—PYCR1, PYCR2, and PYCRL—PYCR1 and PYCR2 are particularly relevant due to their mitochondrial localization and high sequence similarity, while PYCRL is cytosolic^38^. Our results indicate that SUIT-2 cells subjected to FTH1 silencing exhibit a significant decrease in *PYCR1* mRNA and protein levels (Fig. 4), whereas those with induced FTH1 overexpression demonstrate a restoration of PYCR1 expression and a corresponding impact on tumor growth (Fig. 5). Notably, introducing a proline supplement to PYCR1-knockdown SUIT-2 cells reversed the changes in FTH1 protein expression but did not affect the mRNA levels of *FTH1* (Fig. 6). This finding suggested that the interaction between FTH1 and PYCR1 modulates pancreatic cancer progression through a regulatory mechanism affecting proline metabolism, possibly through a posttranslational mechanism that influences the stability of the FTH1 protein. The involvement of proline metabolism in cancer progression was further elucidated by examining the effects of proline inhibitors and supplements. The use of proline inhibitors led to a decrease in cellular proliferation and viability (Fig. 5), reinforcing the importance of proline synthesis for cancer cell growth. Conversely, proline supplementation mitigated the effects of PYCR1 downregulation, partially rescuing the decrease in cell viability and suggesting a potential therapeutic strategy for counteracting the metabolic vulnerabilities of pancreatic cancer cells (Fig. 6). Furthermore, neither PYCR2 nor PRODH protein expression was affected by PYCR1 knockdown (Fig. 6), suggesting that the reduction in FTH1 expression in SUIT-2 cells resulting from PYCR1 knockdown does not involve the activities of PYCR2 or PRODH.

On the basis of the results of Xu et al.^24^, we regarded *miR-1253, miR-6081*, *miR-3150a-3p*, *miR-2355-5p*, and *miR-5000-3p* as the five candidate miRNAs potentially targeting *PYCR1*; our study revealed a nuanced role for miR-2355-5p and miR-5000-3p in the context of pancreatic cancer. We observed that while miR-2355-5p and miR-5000-3p expression was generally downregulated in pancreatic cancer tissues, FTH1 knockdown in SUIT-2 cells led to the upregulation of miR-2355-5p and miR-5000-3p (Fig. 7). These findings imply that elevated levels of these miRNAs, resulting from reduced FTH1, may function as negative regulators of *PYCR1* expression. This finding supports our hypothesis that FTH1 influences PYCR1 expression through the modulation of specific miRNAs, thus contributing to tumor progression in human pancreatic carcinoma. Moreover, inhibitors of miR-5000-3p further confirmed their regulatory role, as the downregulation of these miRNAs led to alterations in *PYCR1* levels, indicating a potential therapeutic target area. Additionally, our analysis of TCGA data revealed a significant correlation between high *FTH1* and *PYCR1* expression and poorer survival outcomes in patients with PDAC, lending clinical relevance to our laboratory findings. In conjunction with miRNA regulation, FTH1 knockdown in PDAC cells was associated with a reduction in intracellular P5C and the expression of collagens I and IV, components critical to the extracellular matrix (ECM). This finding suggests a broader role for FTH1 in ECM remodeling within the tumor microenvironment, a process that is pivotal for cancer cell invasion and metastasis. The TCGA data complement our results, indicating that the concurrent overexpression of the *FTH1* and *PYCR1* genes could be an indicator of aggressive disease and a poor prognosis. Further investigations are warranted to delineate whether FTH1–PYCR1 crosstalk directly contributes to the production of the collagen matrix and, by extension, influences the aggressive behavior of pancreatic cancer. Such studies are essential for understanding the full scope of the impact of FTH1 and PYCR1 on PDAC progression and their potential as targets for novel therapeutic strategies.

Deferasirox (DFX), an FDA-approved oral iron chelation agent, is commonly utilized for the treatment of chronic iron overload due to blood transfusions^39,40^. As a tridentate ligand, DFX binds with high affinity to trivalent iron (Fe^3+^), forming a complex where two DFX molecules coordinate with one Fe3^+^ ion^39^. The antiproliferative effects of DFX on pancreatic cancer cells were initially revealed by Harima et al., who demonstrated that DFX not only hampers pancreatic cancer growth in BALB/c nude mice with BxPC-3 xenografts but also diminishes serum ferritin (SF) levels in vivo^25^. Furthermore, Kim et al. reported a reduction in SF in patients with transfusional iron overload following DFX treatment, suggesting a potential correlation between the efficacy of DFX and SF levels^41^. Nonetheless, the mechanisms underlying the cancer-suppressive actions of DFX, particularly its interactions with ferritin, remain to be elucidated. Consistent with previous findings, our study demonstrated that treatment with DFX significantly impaired the viability of PDAC cells. Notably, this antiproliferative effect of DFX was potentiated following FTH1 knockdown, with SUIT-2/shFTH1 cell viability markedly reduced upon DFX exposure and partially restored with re-expression of FTH1. Furthermore, treatment with DFX substantially decreased FTH1 and FTL protein levels, indicating that DFX may exert its inhibitory effects on pancreatic cancer cell growth through the downregulation of FTH1 activation. The in vivo results of our study further support these in vitro findings. In animal models, FTH1 knockdown paralleled the in vitro results, demonstrating suppressed tumor growth and suggesting an enhancement of the therapeutic effect of DFX. Additionally, the PYCR1/PRODH expression ratio decreased following treatment with DFX, alluding to a DFX-induced alteration in proline metabolism, possibly via FTH1 suppression. Overall, the results from our current study reinforce the notion that DFX possesses considerable therapeutic potential against pancreatic cancer. The suppression of FTH1 not only impaired cancer cell viability but also appeared to sensitize the cells to DFX treatment, thus enhancing the efficacy of the drug. This highlights the dual benefit of directly targeting FTH1-reducing cell viability and increasing susceptibility to further treatment. Consequently, these findings suggest that inhibition of FTH1, possibly through the use of DFX or similar agents, could be a viable strategy to potentiate therapeutic outcomes in pancreatic cancer.

In this study, we elucidated new mechanistic pathways highlighting the interplay between FTH1 and PYCR1, which appears to form a self-reinforcing loop that collectively drives the progression of *KRAS*-mutant pancreatic cancer. Our findings suggest that the interaction between FTH1 and PYCR1 leads to aberrant proline metabolism, subsequently inducing apoptosis in *KRAS*-mutant pancreatic cancer cells. A noteworthy aspect of our research is the regulatory effect of FTH1 on PYCR1 expression, which appears to be mediated through the modulation of miR-5000-3p. The precise mechanisms by which PYCR1 regulates FTH1 expression in PDAC cells remain an area for future research. Crucially, this finding also demonstrated that DFX exerts its antiproliferative effects on pancreatic cancer cells by inhibiting FTH1 expression, suggesting a novel role for DFX in the modulation of proline metabolism. This insight opens up potential avenues for exploiting the therapeutic capabilities of DFX in treating patients with PDAC. Given these observations, the suppression of FTH1 and the subsequent impact of DFX on proline metabolic pathways warrant further exploration as a promising therapeutic strategy in the context of pancreatic cancer.

## Supplementary information

Supplementary Information
