# Author Correction: Crosstalk between FTH1 and PYCR1 dysregulates proline metabolism and mediates cell growth in *KRAS*-mutant pancreatic cancer cells

**DOI:** 10.1038/s12276-025-01437-w

**Published:** 2025-03-27

**Authors:** Ji Min Park, Yen-Hao Su, Chi-Shuan Fan, Hsin-Hua Chen, Yuan-Kai Qiu, Li-Li Chen, Hsin-An Chen, Thamil Selvee Ramasamy, Jung-Su Chang, Shih-Yi Huang, Wun-Shaing Wayne Chang, Alan Yueh-Luen Lee, Tze-Sing Huang, Cheng-Chin Kuo, Ching-Feng Chiu

**Affiliations:** 1 Graduate Institute of Metabolism and Obesity Sciences, Taipei Medical University, Taipei, Taiwan; 2 School of Nutrition and Health Sciences, Taipei Medical University, Taipei, Taiwan; 3 Institute of Cellular and System Medicine, National Health Research Institutes, Zhunan, Taiwan; 4 Division of General Surgery, Department of Surgery, Shuang Ho Hospital, Taipei Medical University, New Taipei City, Taiwan; 5 Department of Surgery, School of Medicine, College of Medicine, Taipei Medical University, Taipei, Taiwan; 6 TMU Research Center of Cancer Translational Medicine, Taipei Medical University, Taipei, Taiwan; 7 National Institute of Cancer Research, National Health Research Institutes, Zhunan, Taiwan; 8 Stem Cell Biology Laboratory, Department of Molecular Medicine, Faculty of Medicine, Universiti Malaya, Kuala Lumpur, 50603 Malaysia; 9 Nutrition Research Center, Taipei Medical University Hospital, Taipei, Taiwan; 10 Department of Bioscience Technology, Chung Yuan Christian University, Taoyuan, Taiwan; 11 Taipei Medical University and Affiliated Hospitals Pancreatic Cancer Groups, Taipei Cancer Center, Taipei Medical University, Taipei, Taiwan

**Keywords:** Pancreatic cancer, Drug development, Diagnostic markers

Correction to: *Experimental & Molecular Medicine* (2024) **56**(9):2065–2081 10.1038/s12276-024-01300-4, published online 18 September 2024

After online publication of this article, the authors noticed an error in the results section and supplementary information.

The correct statement of this article should have read as below.

Modification of language around FTH1 and PYCR1 interaction in the paragraph of Result, under the title of **“FTH1-PYCR1 crosstalk mediates pancreatic cancer progression”** on pages 2071-2073. We would like to clarify of experimental findings supporting the feedback mechanism between FTH1 and PYCR1.

1. **Original text:** “An additional decrease was observed with the overexpression of PYCR1 in clone #4 (Fig. 5b), suggesting a feedback mechanism that modulates FTH1 expression in response to PYCR1 levels. As shown in Fig. 5c, a significant decrease in the PYCR1 mRNA level upon FTH1 knockdown further supports the posttranscriptional regulation of PYCR1 by FTH1. This regulation does not extend to PRODH, as its mRNA levels were not significantly affected by FTH1 knockdown. Cell viability assays revealed that suppression of FTH1 resulted in a significant decrease in the viability of SUIT-2 cells, which was exacerbated by the overexpression of PYCR1 (Fig. 5d), highlighting the role of FTH1–PYCR1 crosstalk in cellular survival.”
  **Revised text:** “Interestingly, the overexpression of PYCR1 in clone #4 rescued FTH1 expression, and conversely, the overexpression of FTH1 rescued PYCR1 expression in these cells, suggesting a positive feedback loop between FTH1 and PYCR1 (Fig. 5b). However, this regulatory mechanism does not extend to PRODH, as its protein levels remained largely unaffected by either FTH1 or PYCR1 overexpression. This distinction highlights a specific interplay between FTH1 and PYCR1 that does not involve PRODH. As shown in Fig. 5c, a significant decrease in the *PYCR1 mRNA* level upon FTH1 knockdown further supports the posttranscriptional regulation of PYCR1 by FTH1. Cell viability assays revealed that suppression of FTH1 resulted in a significant decrease in the viability of SUIT-2 cells, while overexpression of either FTH1 or PYCR1 partially rescued this effect (Fig. 5d), highlighting the role of FTH1–PYCR1 crosstalk in cellular survival.”
2. **Original text:** “This reduction was specific to PYCR1, as the protein levels of PRODH, PYCR2, and FTL remained unchanged. Intriguingly, an increase in FTH1 protein levels was observed in PYCR1-deficient cells (Fig. 6a).”
  **Revised text:** “This reduction was specific to PYCR1, as the protein levels of PRODH and PYCR2 remained unchanged. Intriguingly, the loss of PYCR1 resulted in a reduction in FTH1 protein levels compared to both Scr and Void controls (Fig. 6a), further emphasizing the reciprocal crosstalk between FTH1 and PYCR1.”

This is correctly stated in the **discussion section** of the manuscript: *"These clinical findings align with our in vitro evidence, suggesting that the FTH1-PYCR1 interaction enhances oncogenic activity in KRAS-mutant PDAC Cells."*

3. **Original text (on page 2081):** “AUTHOR CONTRIBUTIONS: C.F.C. led the project oversight. The study was conceptualized, and its design was developed by C.F.C. and J.M.P. The execution of the research and experiments was carried out by J.M.P., Y.H.S., H.H.C., and Y.K.Q. Animal studies were conducted by J.M.P., C.S.F., L.L.C., and Y.K.Q. Data analysis and interpretation were completed by J.M.P., Y.H.S., and Y.K.Q. Clinical sample collection and data analysis were performed by Y.H.S. and H.A.C. The initial draft of the manuscript was written by J.M.P., Y.H.S., and C.F.C., with revisions and editing contributed by J.M.P., C.F.C., T.S.R., J.S.C., S.Y.H., W.S.W.C., A.Y.L.L., and C.C.K. All authors have read and given their approval for the final version of the manuscript.”
  **Revised text:** “AUTHOR CONTRIBUTIONS: C.F.C. and C.C.K. led the project oversight. The study was conceptualized, and its design was developed by C.F.C., C.C.K. and J.M.P. The execution of the research and experiments was carried out by J.M.P., Y.H.S., H.H.C. and Y.K.Q. Animal studies were designed and conducted by J.M.P., Y.K.Q., C.S.F., L.L.C. and T.S.H. Data analysis and interpretation were completed by J.M.P., Y.H.S., and Y.K.Q. Clinical sample collection and data analysis were performed by Y.H.S. and H.A.C. The initial draft of the manuscript was written by J.M.P., Y.H.S., C.F.C. and C.C.K., with revisions and editing contributed by J.M.P., C.F.C., T.S.R., J.S.C., S.Y.H., W.S.W.C., A.Y.L.L., T.S.H. and C.C.K. All authors have read and given their approval for the final version of the manuscript.”
4. **Figures in the original manuscript:**
  **Supplementary Fig. 4e**
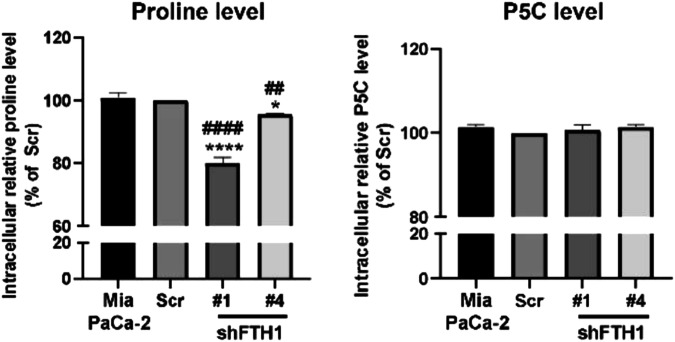
  **Figure legend:** Supplementary Fig. 4e, Intracellular proline and P5C (pyrroline-5-carboxylate) levels in Mia PaCa-2 cells with scrambled shRNA (Scr) and FTH1 knockdown clones #1 and #4 (shFTH1#1, shFTH1#4), showing a significant decrease in proline with FTH1 silencing.
  **Figures after the changes:**
  **Supplementary Fig. 4e**
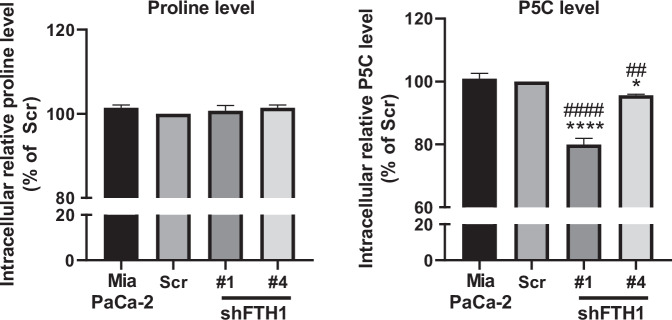
  **Figure legend:** Supplementary Fig. 4e, Intracellular proline and P5C (pyrroline-5-carboxylate) levels in Mia PaCa-2 cells with scrambled shRNA (Scr) and FTH1 knockdown clones #1 and #4 (shFTH1#1, shFTH1#4), showing a significant decrease in P5C with FTH1 silencing.

The authors apologize for any inconvenience caused.

The original article has been corrected.

