# Supplementary material for: Crosstalk between FTH1 and PYCR1 dysregulates proline metabolism and mediates cell growth in KRAS-mutant pancreatic cancer cells
Source: Exp Mol Med. 2024 Sep 18;56(9):2065–81. doi: 10.1038/s12276-024-01300-4 (PMC11447051; doi:10.1038/s12276-024-01300-4)
Supplement: Supplementary file 1 — Supplementary Information [file 12276_2024_1300_MOESM1_ESM.pdf]

Additional file for

Crosstalk between FTH1 and PYCR1 dysregulates proline metabolism and mediates cell growth in *KRAS*-mutant pancreatic cancer cells

Ji Min Park[1,2,3], Yen-Hao Su[4,5,6], Chi-Shuan Fan[7], Hsin-Hua Chen[1], Yuan-Kai Qiu[1,2], Li-Li Chen[7], Hsin-An Chen[4,5,6], Thamil Selvee Ramasamy[8], Jung-Su Chang[1,2,9], Shih-Yi Huang[1,2], Wun-Shaing Wayne Chang[7], Alan Yueh-Luen Lee[7], Tze-Sing Huang[7], Cheng-Chin Kuo[3,10*] and Ching-Feng Chiu[1,6,9,11*]

[1] Graduate Institute of Metabolism and Obesity Sciences, Taipei Medical University, Taipei, Taiwan. [2] School of Nutrition and Health Sciences, Taipei Medical University, Taipei, Taiwan. [3] Institute of Cellular and System Medicine, National Health Research Institutes, Zhunan, Taiwan. [4] Division of General Surgery, Department of Surgery, Shuang Ho Hospital, Taipei Medical University, New Taipei City, Taiwan. [5] Department of Surgery, School of Medicine, College of Medicine, Taipei Medical University, Taipei, Taiwan. [6] TMU Research Center of Cancer Translational Medicine, Taipei Medical University, Taipei, Taiwan. [7] National Institute of Cancer Research, National Health Research Institutes, Zhunan, Taiwan. [8] Stem Cell Biology Laboratory, Department of Molecular Medicine, Faculty of Medicine, Universiti Malaya, 50603, Kuala Lumpur, Malaysia. [9] Nutrition Research Center, Taipei Medical University Hospital, Taipei, Taiwan. [10] Department of Bioscience Technology, Chung Yuan Christian University, Taoyuan, Taiwan. [11] Taipei Medical University and Affiliated Hospitals Pancreatic Cancer Groups, Taipei Cancer Center, Taipei Medical University, Taiwan.

* Corresponding author at: Dr. Ching-Feng Chiu, Graduate Institute of Metabolism and Obesity Sciences, Taipei Medical University, Taipei, Taiwan, E-mail: chiucf@tmu.edu.tw; Dr. Cheng-Chin Kuo, Institute of Cellular and System Medicine, National Health Research Institutes, Zhunan, Taiwan, E-mail: kuocc@nhri.org.tw

Supplementary Materials and Methods

Cell culture

Human nonmalignant pancreatic epithelial (hTERT-HPNE) and human PDAC [BxPC-3, AsPC-1, Mia PaCa-2, SUIT-2, PANC-1, and PANC-1/gemcitabine resistance (GR)] cell lines were kindly provided by Drs. Wun-Shaing Wayne Chang and Li-Tzong Chen from National Institute of Cancer Research (NHRI, Miaoli, Taiwan), whereas the human embryonic kidney cell line HEK293T was purchased from Bioresource Collection and Research Center (Hsinchu, Taiwan). The hTERT-HPNE cells were cultured in Dulbecco's modified Eagle's medium (DMEM) Low Glucose containing 5% fetal bovine serum (FBS), 1% penicillin–streptomycin (PS), and 10 ng/mL epidermal growth factor (EGF). BxPC-3, PANC-1, PANC-1/GR, SUIT-2, and AsPC-1 cells were maintained in Roswell Park Memorial Institute (RPMI) 1640 medium containing 10% FBS and 1% PS. Finally, Mia PaCa-2 cells were maintained in DMEM High Glucose containing 10% FBS, 1% PS, and 2.5% horse serum. DesPanc03, a primary mouse pancreatic cancer cell line, was established from a 12-month-old *LSL-Kras*^{G12D}*/Pdx1*^{cre} (KC) mouse exhibiting a highly fibrotic form of pancreatic ductal adenocarcinoma (PDAC). To ensure optimal growth and viability, the cells were cultured in DMEM High Glucose, supplemented with 10% FBS and 1% PSG (#10378016, Thermo). This medium composition provides the necessary nutrients and

growth factors, along with protection against bacterial contamination, facilitating the maintenance of the cell line under standard cell culture conditions. The cells were cultured under conditions of 37°C and 5% CO2 in a humidified incubator. They were verified to be mycoplasma-free, and their identities were authenticated through STR profiling conducted by both the Bioresource Collection and Research Center (Hsinchu, Taiwan) and the Center for Genomic Medicine at NCKU (Tainan, Taiwan). The culture media were routinely refreshed every three days to sustain optimal growth conditions, and the cultures were allowed to reach 80-90% confluency before proceeding with subsequent experimental manipulations.

***In-vivo* tumor formation assay**

In the *in-vivo* tumor formation assay involving xenograft models, male NOD.CB17-*Prkdc*^{scid}/JNarl (NOD/SCID) mice, aged 8-12 weeks and sourced from the National Laboratory Animal Center (NLAC, Taiwan), were utilized for the study of tumor growth dynamics. Cells from SUIT-2 lines, including the controls (SUIT-2/shScr and shVoid) and the SUIT-2/shFTH1 (targeting knockdown of shFTH#1 and shFTH1#4), were prepared at a concentration of 5×10^5 cells per 100 µL in PBS. This suspension was then mixed equally with Matrigel (BD Biosciences) and administered subcutaneously into the dorsal flank of the mice. Tumor volumes, determined by the formula (length × width^2)/2, were recorded bi-daily once they reached approximately

100–150 mm^3. On the 12th day, tumors were excised and weighed to assess the impact of FTH1 knockdown on xenograft tumor growth. All procedures were conducted in strict adherence to the ethical guidelines set by the Taipei Medical University Animal Care and Use Committee (Approval Nos. LAC-2019-0418 and LAC-2019-0426).

For the evaluation of syngeneic tumorigenesis, C57BL/6 mice (aged 8–12 weeks, NLAC, Taiwan) received a pre-treatment of PBS or Deferasirox (DFX, 160 mg/Kg) via oral gavage. Two days post-pre-treatment, 1×10^6 of DesPanc03 mouse pancreatic cancer cells, originating from a spontaneously derived PDAC model in the *LSL-Kras*^{G12D}/*Pdx1*^{cre} (KC) mouse line and provided by Dr. Tze-Sing Huang from the National Health Research Institutes (NHRI), were implanted subcutaneously in the mice. Following implantation, DFX treatments continued every three days via gavage, with tumor dimensions measured externally using a Vernier caliper and volumes calculated using the formula (length × width^2)/2. The experiment was concluded on the 21st day with the euthanasia of the mice. This study received approval from the Institutional Animal Care and Use Committee of the NHRI (Protocol No: NHRI-IACUC-109196A).

H&E staining and Masson's trichrome staining

Histological analyses were conducted through Hematoxylin and Eosin (H&E) staining and Masson's Trichrome staining. Mouse tissue sections underwent deparaffinization in xylene, followed by rehydration through a graded series of ethanol dilutions. Masson's Trichrome staining was executed employing a Trichrome Stain Kit (ScyTek Laboratories Inc.), strictly adhering to the provided manufacturer's instructions. Subsequently, the stained tissue sections were subjected to a dehydration process utilizing a sequence of graded ethanol solutions, followed by a final treatment in xylene. The preparations were then mounted using a suitable mounting medium at ambient temperature, ensuring preservation and clarity for microscopic examination.

Immunohistochemical staining

Tumor tissues of patients with pancreatic cancer were obtained from Taipei Medical University Joint Biobank (Taipei, Taiwan), and PDAC specimens were diagnosed and subjected to pathologic staging and histologic grading at Taipei Medical University Joint Biobank according to the tumor–node–metastasis (TNM) staging system, provided in Union for International Cancer Control’s *The TNM Classification of Malignant Tumours, 8th Edition* [1]. This study was approved by the Institutional Review Board of Taipei Medical University (No. N20210365 and N202201104), and all patients gave their written informed consent. These were surgical specimens fixed in formalin and embedded in paraffin and then archived. All these archived specimens

were analyzed for FTH1 expression using the IHC staining kit VECTASTAIN Elite ABC HRP Kit (Vector Laboratories), according to the manufacturer's instructions.

Next, we investigated FTH1 expression in the *LSL-Kras*$^{G12D/}$*Pdx1*^{cre} (KC) mouse model mimicking *K-RAS* mutant-driven PDAC development and progression in humans. KC mice develop acinar-to-ductal metaplasia and pancreatic intraepithelial neoplasia (PanIN) in 3~6 months after birth and invasive PDAC in at 6 months of age, exhibiting similar pathological lesions observed in patients with PDAC. To explore the protein expression levels of FTH1, PYCR1, collagen I, and collagen IV throughout the progression of PDAC, IHC staining was conducted on pancreatic tissue sections obtained from KC mice and their syngeneic tumorigenesis counterparts. These sections were probed with antibodies against FTH1 (1:100 dilution, #11682-1-AP, Proteintech Group, Inc), PYCR1 (1:100, #20962-1-AP, Proteintech Group, Inc), collagen I (1:100, #ab34710 and #ab21286, Abcam), and collagen IV (1:100, #ab6586, Abcam). Samples were systematically collected from KC mice at various ages, with five animals representing each time point, to assess temporal changes in protein expression. The use of pancreas tissues from KC mice were conducted in Dr. Tze-Sing Huang's laboratory, adhering to ethical guidelines and with approvals from the Institutional Animal Care and Use Committee of the National Health Research Institutes (Protocol numbers NHRI-IACUC-109196A).

Lentiviral knockdown and *FTH1* and *PYCR1* construct overexpression

shRNAs carrying a puromycin selection marker was purchased from National RNAi Core Facility of Academic Sinica (Taipei, Taiwan). For the establishment of knockdown and control cells, shFTH1 plasmids (shFTH1#1, 5′-GCTCTACGCCTCCTACGTTTA-3′ TRCN0000419951NM_002032; shFTH1#3, 5′-TGCAATGGAGTGTGCATTACA-3′ TRCN0000428722NM_002032; shFTH1#4, 5′-GTGCCGTTGTTCAGTTCTAAT-3′ TRCN0000415027NM_002032), shPYCR1 plasmids (shPYCR1#1, 5′-CACAGTTTCTGCTCTCAGGAA-3′, TRCN0000038979; shPYCR1#2, 5′-TGAGAAGAAGCTGTCAGCGTT-3′ TRCN0000038983), and control [shScramble (Scr), 5′-CCTAAGGTTAAGTCGCCCTCG-3′; shVoid (Void), N/A] plasmids were generated in HEK239T cells using pCMV deltaR8.91 and pCMV-VSV-G. Cell culture supernatants containing lentivirus particles were then collected and used to infect either SUIT-2 or Mia PaCa-2 cells in the presence of 8 μg/mL polybrene (TR-1003, Merck). After 48 hr, the stable cells were selected with 2–4 μg/mL puromycin and pooled for subsequent analyses.

To visualize the rescue effect of FTH1 in SUIT-2/shFTH1 cells, FTH1 overexpression plasmid (p7126 pHAGE-P-CMVt-N-HA-GAW-FTH1; #100152, Addgene,) was used; the appropriate lentivirus particles were generated in HEK293T cells and used to infect SUIT-2/shFTH1 cells (shFTH#1 and shFTH#4), as described

previously. For PYCR1-overexpressing cells, the SUIT-2/shFTH1 cells were transiently transfected with the plasmids encoding PYCR1, gifted by Alan Yueh-Luen Lee, along with 0.2 μg of pCMV-AC-green fluorescent protein (GFP) by using the transfection reagent DreamFect Gold (DG80500; OZBiosciences).

Ras activation

HEK293T cells were seeded in 60-mm-diameter dishes and incubated overnight. On the following day, the cells were cotransfected with plasmids expressing pUSE-Ampicilin (empty vector, Clontech), pCMV-*RAS*-wild-type (WT, #K6013-1, Clontech), pCMV-*RAS*-constitutively active (V12, #K6013-1, Clontech), or pCMV-*RAS*-dominant-negative (N17, #K6013-1, Clontech), along with pCMV-AC-green fluorescent protein (GFP) using DreamFect Gold. After 48 hr of incubation, the cells were collected for Western blotting.

Western blotting

All the collected cells were lysed using RIPA buffer with a protease inhibitor cocktail (4906845001; Merck) and phosphatase inhibitor (TAAR-BB13; BioTools). By using a Bio-Rad protein assay kit, we determined protein concentrations; equal amounts of proteins were separated through sodium dodecyl sulfate polyacrylamide gel electrophoresis (SDS-PAGE) and then transferred onto a PVDF membrane. After

blocking them with 5% skim milk in Tris-buffered saline containing 0.1% Tween-20 (TBST) for 30 min, we incubated the membranes with primary antibodies against β-actin (1:7500; #A1978, Millipore), α-tubulin (1:7500; #A6830, ABclonal), GAPDH (1:7500; #A10868, ABclonal), pERK1/2 (1:1000; #9101, Cell Signaling Technology), total Erk1/2 (1:2000; #E-AB-12397, Elabscience), FTH1 (1:2500; #CPA4412, Cohesion), FTL (1:500; #LS-C212054, LSBio), PYCR1 (1:7500; #20962-1-AP, Proteintech), PYCR2 (1:7500; #CQA5644, Cohesion), and PRODH (1:2000; #CQA1282, Cohesion) overnight. The membranes were then washed with TBST for 5 min three times, followed by incubation with appropriate horseradish peroxidase–conjugated secondary antibodies at room temperature for 1 h. Protein bands were detected using the electrochemiluminescence reagent (WBLUF0500; Millipore) and imaged and assessed using the UVP biochemical system and VisionWorks LS (VisionWorks).

RNA extraction and qRT-PCR

Total RNA was isolated using NucleoZOL (740404.200; Macherey-Nage) and employed as a template for reverse transcription to cDNA by using the M-MLV reverse transcription kit (18057018; Invitrogen), according to the manufacturer’s instructions. In brief, qRT-PCR was performed using the Diamond 2× Q-PCR Master Mix (GB1100, GoalBio, Taiwan) on a Roche LightCycler 480 PCR platform (Roche Molecular

Systems, USA). The sequences of the primers utilized in this study are detailed in Supplementary Table 1. All the primers were designed using Roche's Universal Probelibrary Assay Design Center. The PCR cycle number (CP) was calculated using LightCycler 480. The relative levels of mRNA expression were normalized to the mean levels of *GAPDH* mRNA. The relative levels of *gene* expression are presented as ΔCP, and the fold change in gene expression was calculated as $2^{-\Delta\Delta CP}$.

Cell viability

The established stable SUIT-2 or Mia PaCa-2 cells were collected and examined for viability by using the 3-(4,5-dimethylthiazol-2-yl)-2,5-diphenyltetrazolium bromide (MTT) assay. The cells were seeded in 96-well plates at a density of 5000 cells per well and incubated at 37°C in 5% CO_2 atmosphere overnight. On the next day, the cells were treated with DFX (#A10293, AB Science), L-proline (#P5607, Sigma-Aldrich), proline analog inhibitor (N-Formyl-L-proline, #13200-83-4, ChemScene) [2], PYCR1 inhibitor (PYCR1-IN-1, #HY-126271, MedChemExpress) , or gemcitabine HCl (#G6423, Sigma-Aldrich) and then incubated for 24, 48 and 72 hr. The MTT reagent was added to a final concentration of 1 μg/μL to each well and incubated for an additional 4 h. Thereafter, the medium was removed completely, followed by the addition of 100 μL of DMSO per well; the plates were shaken gently at room temperature for 10 min to

dissolve the MTT–formazan crystals. The absorbance was measured at 570 nm on the EPOCH2 microplate reader.

Colony formation assay

To assess the long-term proliferative capacity, a clonogenic assay was conducted by inoculating 200 cells per well in a 6-well culture dish, with media replenishment occurring triennially. Subsequent to an incubation period ranging from 8 to 10 days, the culture medium was aspirated, and the resultant cell colonies were subjected to fixation using a 10% formalin solution for a duration of 10 minutes. Following fixation, the colonies underwent staining with a 0.5% crystal violet solution prepared in methanol for 10 minutes, followed by thorough rinses in distilled water. Digital imaging equipment was utilized to capture visual records of the colonies, and quantitative analysis of colony formation was executed utilizing the ImageJ software platform.

Flow cytometry analysis

For cell-cycle analysis, 1×10^6 of cell suspension was centrifuged at 1500 rpm at room temperature for 10 min. The supernatants were then removed and washed with ice-cold PBS; this was followed by dropwise addition of 1 mL of 70% cold ethanol for fixing the cells. Cells were permeabilized at −20°C overnight and then washed again with PBS. Next, the staining mixture, containing 20 µg/mL propidium iodide (PI), 10% Triton X-100, and 10 µg/mL RNase A, prepared in PBS, were added. After re-suspension, cells

were incubated at 37°C for at least 30 min and analyzed using an Attune Nxt flow cytometer (Invitrogen) and Flowjo.

Global metabolic profiles

Global metabolic profiles were screened and analyzed among two groups: SUIT-2/shCtrl and SUIT-2/shFTH1. The association of metabolic pathways with FTH1 in SUIT-2 metastatic pancreatic cancer cells was mapped to determine the potential metabolic pathway involved in FTH1 regulation. In brief, 1×10^6 cells were cultured in RPMI 1640 containing 10% FBS for 3 days; thereafter, 10 mL of cultured medium was collected in 40 mL of methanol, followed by incubation at room temperature for 1 h with gentle shaking and then addition of 1.25 mL of H_2O. Next, this mixture was centrifuged at 3000 rpm for 10 min at 4°C, and the supernatant was removed. The collected bottom layer was then vacuum dried and stored at −80°C until analysis.

Liquid chromatography–tandem mass spectrometry (LC–MS/MS) experiments of all extracts were performed using an ACQUITY UPLC CSH C18 column on a SYNAPT G2 HDMS mass spectrometer (Waters, Milford, MA, USA) at the Mass Spectrum Core Facility of Taipei Medical University. The Alliance LC system and Q-TOF micro instrument was controlled using MassLynx (version 4.0; Waters). Raw LC–MS data was further processed using MarkerLynx, a metabolomics application widely used for data processing. The data analysis software program SIMCA-P (Umetrics,

Umea, Sweden) was used for some aspects of multivariate data processing. The metabolites identified as candidates in the analysis are enumerated in Supplementary Table 2.

Glutamine consumption and glutamate secretion assay

The quantification of extracellular and intracellular levels of glutamine and glutamate in SUIT-2/shScr, SUIT-2/shVoid, SUIT-2/shFTH#1, and SUIT-2/shFTH1#4 cell lines was performed utilizing the Glutamine/Glutamate-Glo assay kit (#8021, Promega), adhering to the protocol specified by the manufacturer. Concisely, 5000 cells were seeded in 96-well plates and allowed to incubate overnight. Subsequently, 2 µL of culture medium was carefully extracted from each well, diluted with 98 µL of PBS, and aliquots of this dilution were combined with 12.5 µL of either Glutaminase (GLS) Buffer or Glutaminase Enzyme Solution. This mixture was then incubated at ambient temperature for a duration of 30 minutes. Following this incubation period, 25 µL of Glutamate Detection Reagent was introduced to each well, and the mixture was incubated once more at room temperature for 60 minutes. The luminescence generated by this reaction was quantitatively measured using a plate-reading luminometer (Thermo).

Measurement of proline and P5C levels

Proline and P5C levels were measured using the ninhydrin method, described by Guo *et al.* and Miller *et al.* [3,4]. For proline level measurement, the cells were transferred to a serum-free medium 1 day before measurement. In total, 4×10^6 cells were lysed with PBST (1% Triton X-100 in PBS) and centrifuged at 9000 g and 4°C for 10 min for cell debris removal. The supernatant was then collected and placed into a boiling water bath for 10 min. After centrifugation at 15,000 g and 4°C for 15 min, 200 µL of supernatant was mixed with 1.25% ninhydrin and incubated at 100°C for 20 min. The absorbance was measured at 520 nm on the EPOCH2 microplate reader.

For P5C level measurement, the cells were transferred to a serum-free medium 1 day before measurement. In total, 4×10^6 cells were lysed with 1 N HCl and centrifuged at 9000 g and 4°C for 10 min for cell debris removal. The supernatant was then collected, and 200 µL of the supernatant was mixed with 1.25% ninhydrin and incubated at 100°C for 20 min. The absorbance was measured at 520 nm on the EPOCH2 microplate reader.

Detection and Inhibition of microRNAs

Sequences of mature miRNAs were sourced from the Sanger Center miRNA Registry (http://microrna.sanger.ac.uk/sequences/), and the design of stem-loop RT primers was guided by the methodology proposed by Chen *et al* [5]. The protocol for detecting mature microRNAs involved real-time RT-PCR reactions, which comprised 0.5 µM of each

forward and reverse primer, 1 μM of Universal ProbeLibrary Probe #21 (Roche), a 1× concentration of LightCycler TaqMan Master mix, and 2 μl of cDNA. The amplification process was initiated with a denaturing phase at 95°C for 10 minutes, followed by 65 cycles of denaturation at 95°C for 5 seconds, annealing at 60°C for 10 seconds, and extension at 72°C for 1 second. The *U47* small nuclear RNA was employed as the reference *gene* in this analysis. The primer sequences used are specified in Supplementary Table 1. Relative gene expression levels were quantified using the ΔCP method, where ΔCP = CP of the target *gene* minus CP of the reference *gene*, and changes in gene expression were calculated employing the $2^{-\Delta\Delta CP}$ formula. The miR-2355-5p and miR-5000-3p antagomirs (inhibitors) along with the miR-NC (miRNA inhibitor control) were procured from PhalanxBio Inc. (Hsinchu, Taiwan), with their application following the manufacturer's guidelines to suppress the expression of targeted miRNAs.

Survival analysis using Kaplan–Meier Plotter and PROGgeneV2

The Kaplan–Meier Plotter (http://kmplot.com/analysis) was used to analyze the correlation between the survival of patients with pancreatic cancer and the expression of genes of interest. Specifically, Kaplan–Meier survival curves were plotted to investigate the correlations of *FTH1, FTL, PYCR1, PYCR2, PYCR3,* and *PRODH* expression with the overall survival of patients with pancreatic cancer. PROGgeneV2

(http://genomics.jefferson.edu/proggene) was used to perform survival analyses on the basis of the expression ratio of any two genes. Survival curves comparing pancreatic cancer patients with high/high (red) and low/low (green) expression of *FTH1/PYCR1* or *FTH1/KRAS* were plotted using PROGgeneV2 to investigate the prognostic significance of the cooccurrence of any two genes.

Clinical pancreatic cancer Samples and Expression Data

Clinical, genomic, and expression data for this study were sourced from the TCGA cohort, accessed through the NIH Genomic Data Commons (GDC). We analyzed mRNA levels of miR-2355-5p and miR-5000-3p in biopsy tissues from 178 pancreatic cancer patients and 4 adjacent non-tumor samples within the cohort. Data are presented as mean ± SEM. To determine the association between these microRNAs and PYCR1 expression in *KRAS*-mutated pancreatic tumors, we isolated cases with KRAS mutations. Spearman's rank correlation test was conducted to assess the relationship, utilizing Spearman correlation coefficients computed in R (v3.6.2) using the cor.test() function, which also provided the 2-tailed *P* values for the correlations.

Statistical analysis

The data are expressed as mean ± standard deviation (SD) or mean ± standard error of the mean (SEM). Graphs were generated, and quantitative results were compared using Student's *t* test. Significant differences between the groups were determined using one-

way analysis of variance (ANOVA) followed by Tukey’s post hoc test. A p of <0.05 was considered to indicate statistical significance. All statistical analysis was performed using Prism (GraphPad, La Jolla, CA, USA).

Supplementary Tables

Supplementary Table 1: Primer Sequences Used in the Study

Gene name	Clone ID or orientation	Sequence 5' →3'
GAPDH	Sense	agccacatcgctcagacac
	Antisense	gcccaatacgaccaaatcc
FTH1	Sense	gccagaactaccaccaggac
	Antisense	catcatcgcggtcaaagtag
FTL	Sense	ctggagaaaaagctgaaccag
	Antisense	tccaggaagtcacagagatgg
PYCR1	Sense	atgagcgtgggcttcatc
	Antisense	cttgtgggcagccaaga
PRODH	Sense	ctaagcccttgtctggcatt
	Antisense	tgagcttgttgaatagcctctg
hsa-miR-2355-5p	Forward primer	cggatcccagatacaatggacaa
	RT sequence	gttggctctggtgcagggtccgaggtattcgcaccagagccaacttgtcc
hsa-miR-5000-3p	Forward primer	gcggtcaggacacttctgaa
	RT sequence	gttggctctggtgcagggtccgaggtattcgcaccagagccaactccaag
U47	Forward primer	cggcggtaatgattctgccaaa
	RT sequence	gttggctctggtgcagggtccgaggtattcgcaccagagccaacacctcag
**Universal reverse primer**	Reverse primer	gtgcagggtccgaggt

Supplementary Table 2: Candidate Metabolites Identified in the Analysis

Metabolic pathway	Candidate metabolites
**Proline metabolism**	L-proline, Guanidinopentanoic acid, 5-oxo-L-proline 2-naphthylamide, 5-oxo-L-proline, 2-Naphthylamine, 1-octadecanoyl-2-(9Z)-9-octadecenoyl-glycerol 3-phosphate, 2-Amino-5-guanidino-pentanoic acid, Lys-Ala 2-naphthylamide, 2-Naphthylamide
**Aminosugar metabolism**	Glucosamine, 2-nitroacetophenol
**Mitochondrial in beta-oxidation of long-chain saturated fatty acids**	Acetyl-CoA, 3-oxo-11,14-eicosadienoyl CoA, 2-Naphthylamine, Acyl-L-carnitine, 1-Oleoyl-2-(9Z)-9-octadecenoyl-glycerol 3-phosphate, 1-Oleoyl-phosphatidic acid (2R), Acyl-L-carnitine, Carnitine, 2-deoxy-alpha-D-ribose 1-phosphate, (2E,9Z)hexadeca dienoyl-CoA, 1-Oleoyl-phosphatic acid (2R), (9Z)-9-octadecenoyl-glycerol-3-phosphate, (E)-Hex-2-enoyloxy alpha cellulose
**2-Naphthylamine and 2-Nitronapgtalene metabolism**	Guanidinopentanoic acid, 5-oxo-L-proline, 2-Naphthylamine, Acyl-CoA, 3-oxo-11,14-eicosadienoyl CoA, 2-Naphthylamine, 2-naphthylamine, 2-Nitroacetophenone
**Galactose metabolism**	3-Formylpyridine
**Thiamine triphosphate (TTP) metabolism**	Thiamine, Thiamine pyrophosphate
**Tyrosine metabolism**	DOPA (3,4-Dihydroxyphenylalanine), Dopamine, Norepinephrine, Epinephrine, Melanin, Homogentisic acid, Fumarate, Succinate, Acetyl-CoA
**Histidine-glutamate-glutamine metabolism**	Proline, Arginine, Formiminoglutamate, α-Ketoglutarate, Glutamine, N-Acetylglutamate

The metabolites listed in this table were identified as being significantly downregulated following the knockdown of FTH1 in SUIT-2 cells, as determined by LC/MS analysis. All metabolites exhibited a fold change greater than 1.5 to 2, with a *P*-value of less than 0.01, indicating significant changes in their levels post-knockdown.

Supplementary Figures

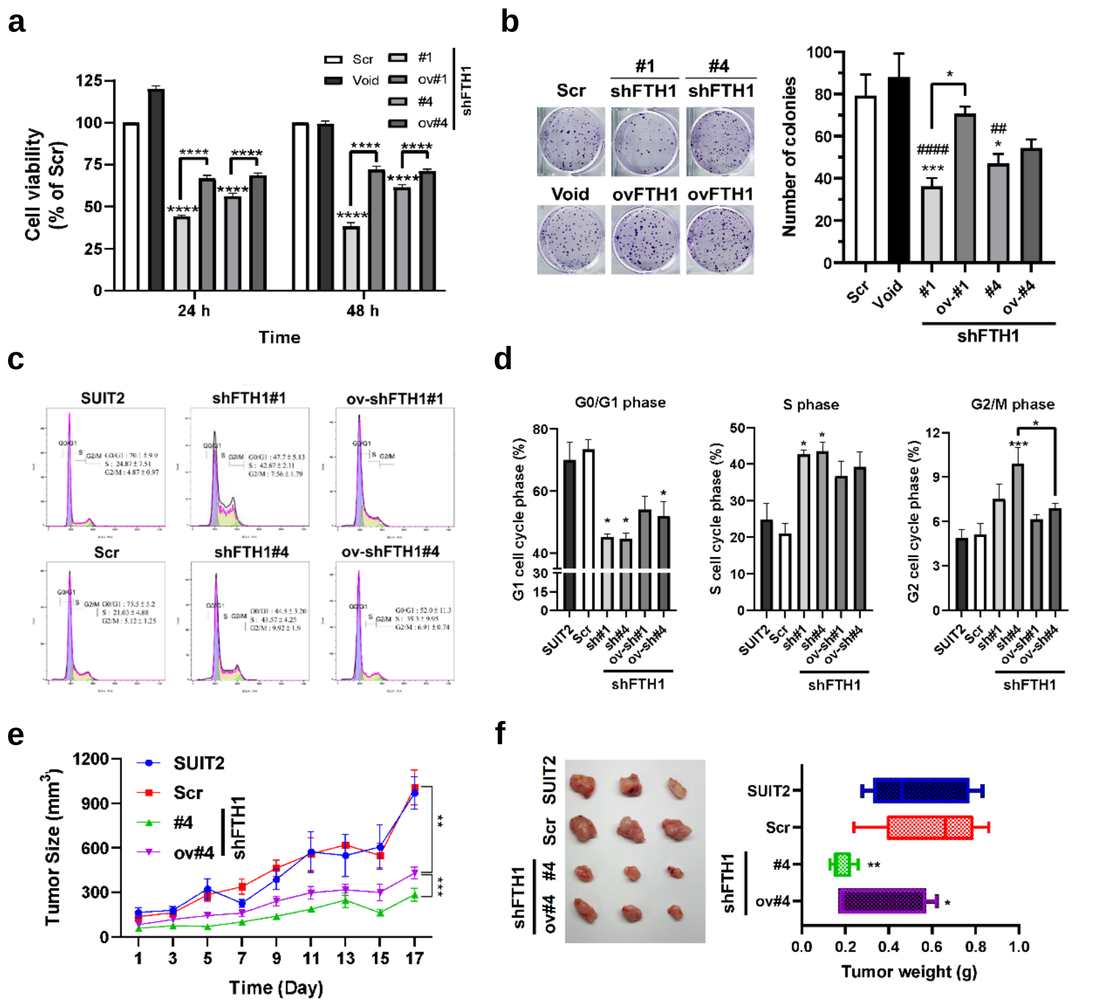

Supplementary Fig. 1: Comprehensive *in vitro* and *in vivo* functional characterization of FTH1 in SUIT-2 cells. a: Cell viability assay at 24 and 48 hours for SUIT-2 cells with scrambled shRNA (Scr), FTH1 knockdown clones #1 and #4 (shFTH1#1, shFTH1#4), and overexpression clones of FTH1 (ovFTH1#1, ovFTH1#4). **b**: Colony formation assay showing the number of colonies for each cell type in the indicated SUIT-2 cells. **c**: Cell cycle analysis of the indicated SUIT-2 cells using flow cytometry with representative histograms. **d**: Quantification of cell cycle distribution, with percentages of cells in G0/G1, S, and G2/M phases. **e**: In vivo tumor growth curves over 17 days post-implantation of the indicated SUIT-2 cells into mice. **f**: Representative images of excised tumors and quantification of tumor weight at the end of the study. Data are represented as mean ± SEM. Significance denoted by *$p < 0.05$, **$p < 0.01$, ***$p < 0.001$, ****$p < 0.0001$ compared to Scr, and #$p < 0.05$, ##$p < 0.01$, ###$p < 0.001$, ####$p < 0.0001$ compared to corresponding shFTH1.

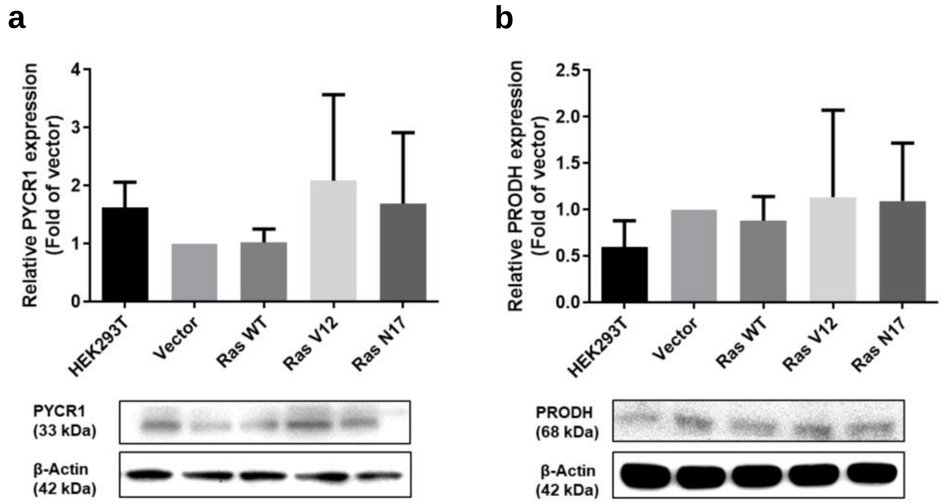

Supplementary Fig. 2: RAS activation states do not modulate PYCR1 and PRODH expression. a and **b**: HEK293T cells were transfected with empty vector, wild-type RAS (WT), constitutively active RAS (V12), or dominant-negative RAS (N17) constructs. Subsequent immunoblots were performed to determine the expression levels of PYCR1 and PRODH, respectively. β-Actin served as a loading control for normalization. Quantitative data presented below the blots show the relative expression of (**a**) PYCR1 and (**b**) PRODH, normalized to the vector control. Results are displayed as means ± SD from three individual experiments (n = 3).

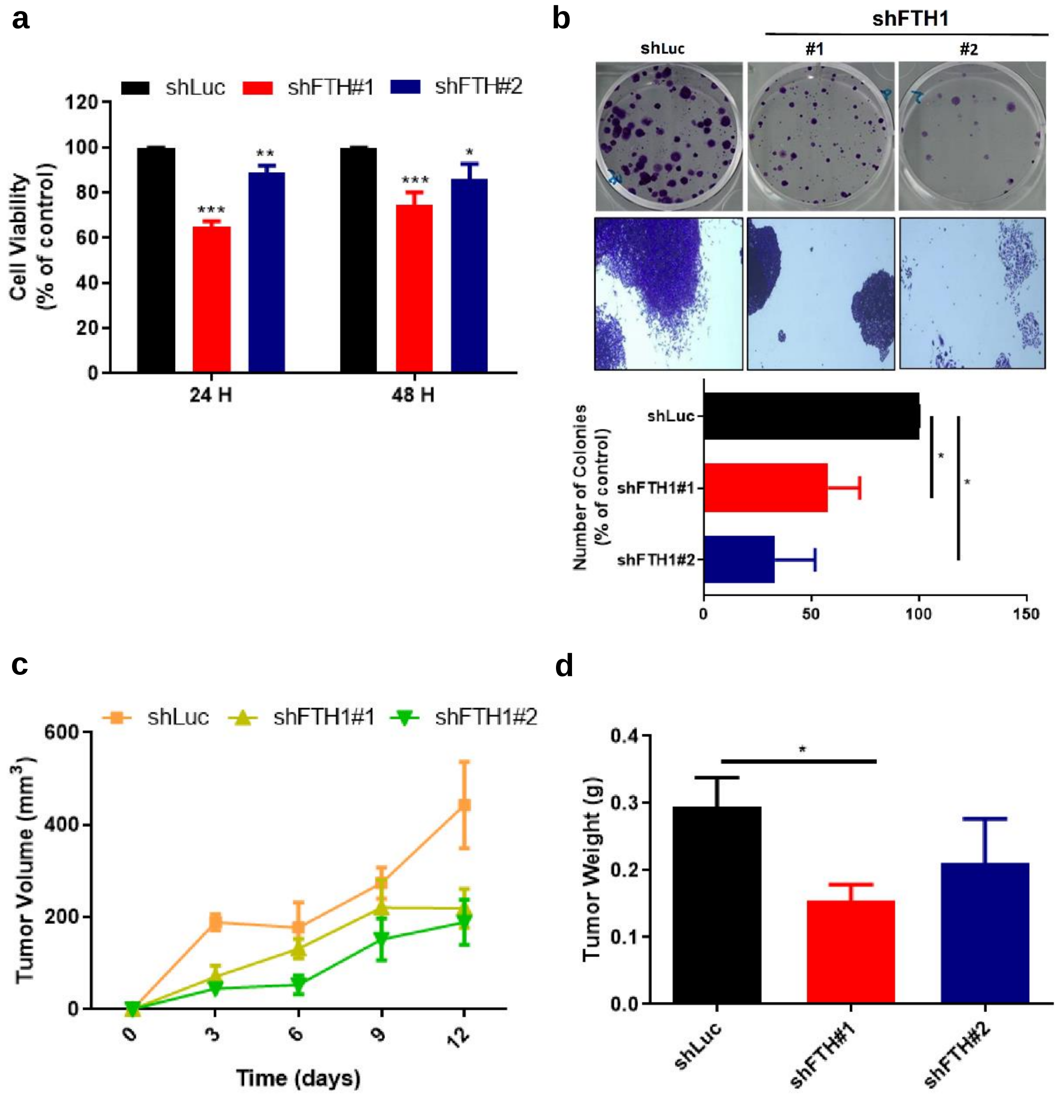

Supplementary Fig. 3: Functional impact of FTH1 knockdown on cell viability, colony formation, and tumor growth. a: Cell viability assay showing percentage of SUIT-2 cells at 24 and 48 hours post-transfection with shRNA targeting luciferase (shLuc) as control and two different shRNAs targeting FTH1 (shFTH1#1 and shFTH1#2). **b**: Colony formation assay with representative images and quantification of colonies indicating the reduced clonogenic potential in FTH1 knockdown SUIT-2 cells. **c**: In vivo tumor growth curves over a 12-day period post-injection with the SUIT-2 cells carrying shLuc, shFTH1#1, or shFTH1#2. **d**: Tumor weight measurements at the endpoint of the in vivo study, showing a decrease in tumor mass in FTH1-silenced groups. Data represent mean ± SEM, with *$p < 0.05$, **$p < 0.01$, ***$p < 0.001$ indicating levels of statistical significance.

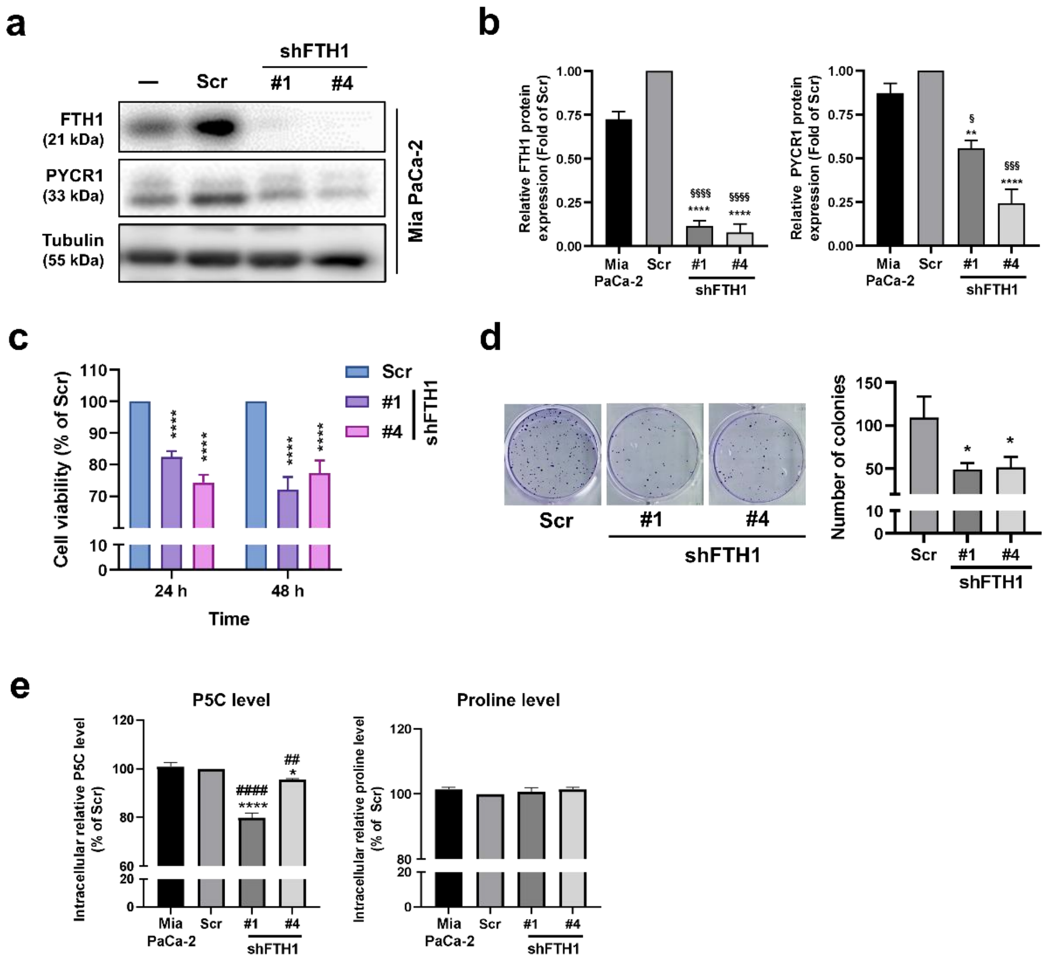

Supplementary Fig. 4: Effects of FTH1 knockdown on proline metabolism, protein expression, cell viability, and colony formation in pancreatic cancer cells. a: Western blots of FTH1 and PYCR1 protein expression, with Tubulin as a loading control. **b**: Densitometric analysis of FTH1 and PYCR1 expression normalized to Mia PaCa-2 cells. **c**: Cell viability assay indicating reduced viability in FTH1 knockdown clones at 24 and 48 hours post-transfection. **d**: Colony formation assay with representative images (left) and quantification (right), demonstrating decreased clonogenicity in shFTH1 cells. **e**: Intracellular proline and P5C (pyrroline-5-carboxylate) levels in Mia PaCa-2 cells with scrambled shRNA (Scr) and FTH1 knockdown clones #1 and #4 (shFTH1#1, shFTH1#4), showing a significant decrease in P5C with FTH1 silencing. Data are expressed as mean ± SEM. Significance is denoted by *p < 0.05, **p < 0.01, ***p < 0.001, ****p < 0.0001 compared to Scr, ##p < 0.01, ####p < 0.0001 compared to Mia PaCa-2, and $^{§§}p < 0.01$, $^{§§§}p < 0.001$, $^{§§§§}p < 0.0001$ compared to respective controls.

a

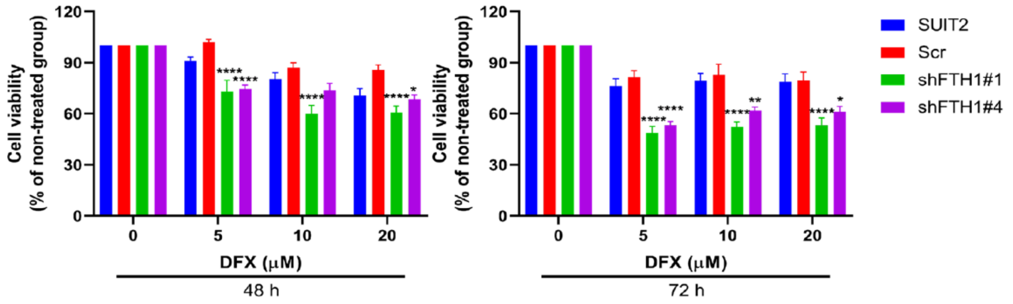

b

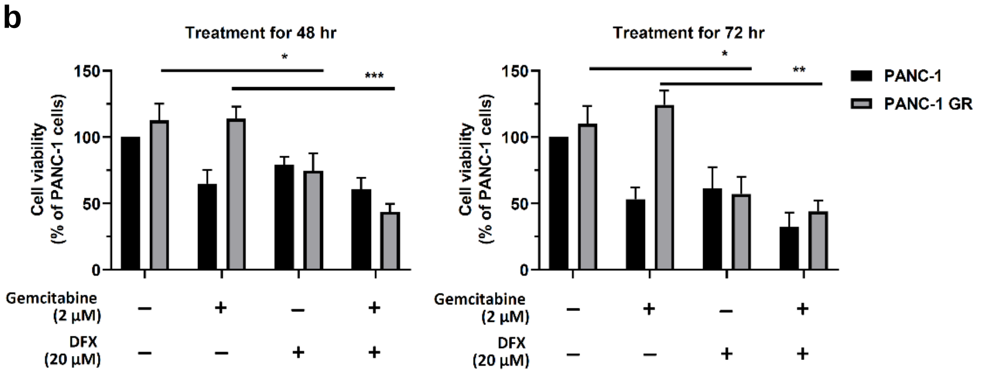

Supplementary Fig. 5: Effect of Deferasirox (DFX) on the viability of pancreatic cancer cells and the enhancement of gemcitabine efficacy. **a**: Cell viability of SUIT-2, scrambled shRNA control (Scr), and FTH1 knockdown cells (shFTH1#1 and shFTH1#4) after 48 and 72 hours of treatment with different concentrations of DFX, showing dose-dependent declines in viability. **b**: Synergistic effects of DFX on gemcitabine-treated PANC-1 and gemcitabine-resistant PANC-1 (PANC-1 GR) cells over 48 and 72 hours, indicating that DFX potentiates the reduction in cell viability by gemcitabine. Viability is expressed as a percentage of non-treated control cells, with data presented as mean ± SEM. Significance is noted as *$p < 0.05$, **$p < 0.01$, and ***$p < 0.001$.

a

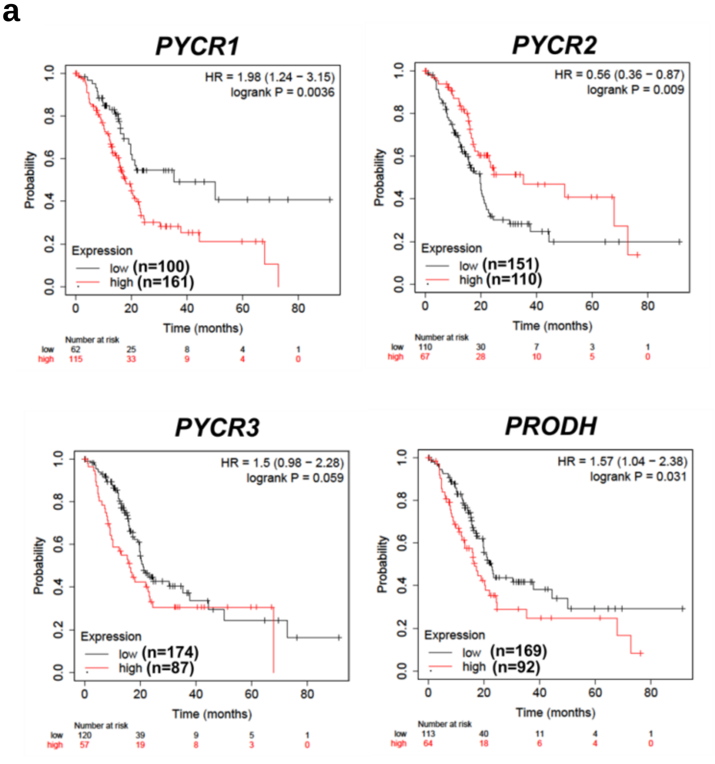

b

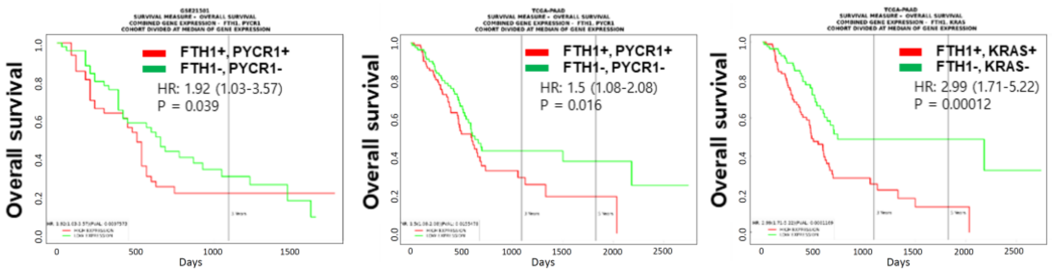

Supplementary Fig. 6: Prognostic impact of FTH1 and PYCR family gene expression in pancreatic cancer patients. a: Kaplan–Meier survival analysis comparing patient outcomes based on gene expression levels. High expression (red line) versus low expression (black line) of PYCR1, PYCR2, PYCR3, and PRODH shows varying hazard ratios (HRs) and prognostic significance. **b**: Survival outcomes based on the combined expression status of FTH1 and PYCR1, demonstrating that co-occurrence (high/high) correlates with poorer survival compared to non-cooccurrence (low/low). Data were analyzed from GSE21501 and TCGA datasets. Plots generated using Kaplan–Meier Plotter and PROGgeneV2 tools, providing hazard ratios and p-values to assess statistical significance.

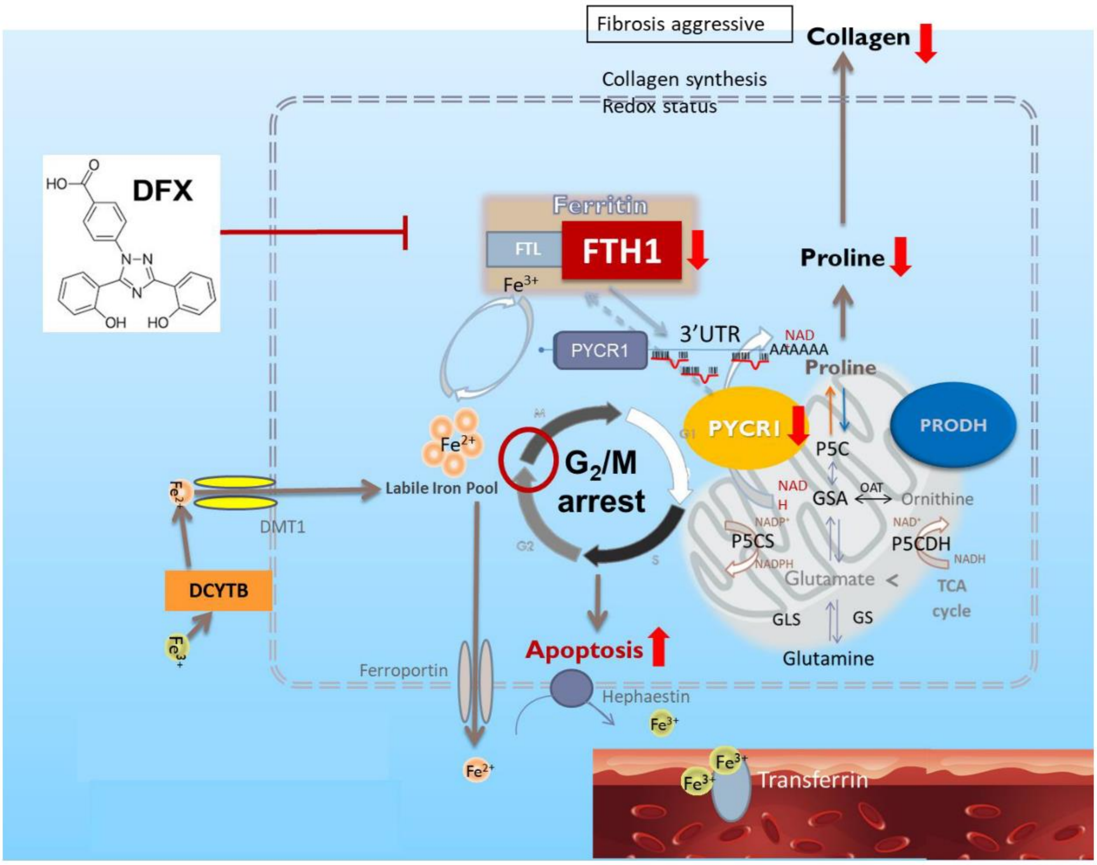

Supplementary Fig. 7: Proposed schematic illustrating the interplay between FTH1 and PYCR1 in the disruption of proline metabolism within pancreatic cancer cells. The model outlines the impact of Deferasirox (DFX) on cellular iron levels and how it leads to a decrease in collagen synthesis and proline levels, potentially through FTH1 inhibition. This disruption is shown to cause cell cycle arrest at G2/M and induce apoptosis. The diagram highlights the downstream consequences of altered iron metabolism, which include changes in the labile iron pool, affecting the activity of key enzymes in the proline and glutamine metabolic pathways.

References:

1 Park, M. Y. *et al.* Validation of the eighth edition of the American Joint Committee on Cancer staging system and proposal of an improved staging system for pancreatic ductal adenocarcinoma. *Ann Hepatobiliary Pancreat Surg* **23**, 46-55, doi:10.14701/ahbps.2019.23.1.46 (2019).

2 Christensen, E. M. *et al.* In crystallo screening for proline analog inhibitors of the proline cycle enzyme PYCR1. *J Biol Chem* **295**, 18316-18327, doi:10.1074/jbc.RA120.016106 (2020).

3 Guo, L. *et al.* PINCH-1 regulates mitochondrial dynamics to promote proline synthesis and tumor growth. *Nat Commun* **11**, 4913, doi:10.1038/s41467-020-18753-6 (2020).

4 Miller, G. *et al.* Unraveling delta1-pyrroline-5-carboxylate-proline cycle in plants by uncoupled expression of proline oxidation enzymes. *J Biol Chem* **284**, 26482-26492, doi:10.1074/jbc.M109.009340 (2009).

5 Chen, C. *et al.* Real-time quantification of microRNAs by stem-loop RT-PCR. *Nucleic Acids Res* **33**, e179, doi:10.1093/nar/gni178 (2005).
